# Sustainable production of ZnO nanoparticles *via capparis decidua* stem extract for efficient photocatalytic Rh 6G dye degradation

**DOI:** 10.1039/d5ra05878c

**Published:** 2025-11-27

**Authors:** Asmaa M. Sayed, Fawzy M. Salama, Hanaa K. Galal, Mohamed I. Said

**Affiliations:** a Department of Botany and Microbiology, Faculty of Science, Assiut University Egypt; b Department of Chemistry, Faculty of Science, Assiut University Egypt moh_chem1@yahoo.com mohamedali123@aun.edu.eg

## Abstract

Zinc oxide nanoparticles (ZnO NPs) were sustainably synthesized using *Capparis decidua* stem extract as a green capping and stabilizing agent, and their photocatalytic efficiency toward Rhodamine 6G (Rh 6G) degradation was evaluated. Three extract concentrations (2, 5, and 10 g per 200 mL) were employed to optimize synthesis and performance. The ZnO NPs were characterized by XRD, FTIR, TEM, UV-Vis spectroscopy, and N_2_ adsorption–desorption analysis. Pristine ZnO NPs were obtained using low plant extract concentrations. TEM revealed spherical nanoparticles whose average diameter decreased from 40 to 24 nm with increasing extract concentration. The sample synthesized with the highest extract concentration exhibited the largest surface area (32.9 m^2^ g^−1^) and pore volume (0.039 cm^3^ g^−1^), indicating the impact of extract concentration on the material texture. The optical band gap narrowed from 3.67 eV to 3.02 eV while increasing the extract concentration from 2 g/200 mL (sample 2Z) to 10 g/200 mL (sample 10Z). Under sunlight irradiation, the 2Z sample achieved the highest photocatalytic degradation efficiency (for Rh 6G) of ∼96% at the optimum pH of 6.5 (in 75 min), compared to ∼88.4% for the sample 10Z. The degradation followed first-order kinetics with a rate constant of 0.018 min^−1^. Superoxide radicals (O_2_˙^−^) were the primary reactive species governing the photocatalytic degradation of Rh 6G, with ˙OH and (h^+^) contributing secondary roles in the overall oxidation. These findings demonstrate the potential of *Capparis decidua* extract for the eco-friendly synthesis of efficient ZnO photocatalysts for environmental remediation.

## Abstract 2

Zinc oxide nanoparticles (ZnO NPs) were sustainably synthesized using *Capparis decidua* stem extract as a green capping and stabilizing agent, and their photocatalytic efficiency toward Rhodamine 6G (Rh 6G) degradation was evaluated.

## Graphical abstract

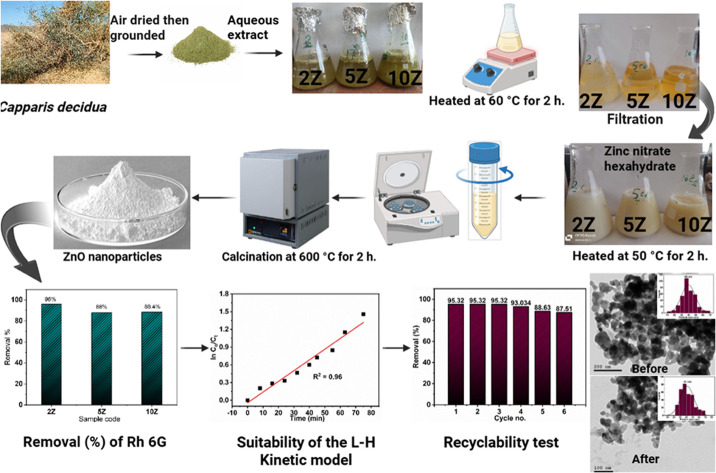

## 1. Introduction

Biosynthesis among the various methods used for synthesis of nanoparticles (NPs) has garnered significant attention from researchers due to its advantages over the traditional techniques.^1,2^ This method is advantageous because it is easy to design, cost-effective, non-toxic, non-volatile, and energy-efficient. A notable benefit is the use of biological extracts, which effectively reduce and stabilize NPs. This approach involves the bio-oxidation or bio-reduction of metal-based precursor salts using extracts from various plant parts or specific bacteria.^3^ Plant extracts, in particular, contain a diverse array of active biomolecules, including proteins, carbohydrates, vitamins, polymers, and natural surfactants, which contribute to the stability of the synthesized NPs.^4^

Various chemical and physical techniques, including laser pyrolysis, spray pyrolysis, sol–gel processes, chemical bath deposition, and chemical vapor deposition, are employed for formation of nanomaterials. However, these methods often rely on toxic chemicals and solvents, and some are energy-intensive due to the high temperatures and pressures required. Additionally, the morphological characteristics of the materials are influenced by the reaction conditions such as chemical concentration, pH, and temperature. Traditional chemical compounds and organic solvents can be hazardous, and managing waste materials can be challenging. In contrast, the green synthesis method reduces the risk of contamination at the source and minimizes waste generation.

Zinc oxide (ZnO) is recognized as an n-type semiconductor, distinguished by its wide band gap of 3.37 eV. Its unique combination of electrical, optical, and chemical properties makes it a promising material for a wide range of industrial and research applications.^5–7^ ZnO is extensively utilized in photonic crystals, light-emitting devices, photodetectors, photodiodes, and solar cells, as well as in the development of highly sensitive gas sensors and biological and chemical sensors.

Herein, stem extracts from *Capparis decidua* were utilized for the biosynthesis of ZnO NPs. *Capparis decidua*, commonly known as Karir, Caper, Han bag, and Karyal, belongs to the *Capparidaceae* family of plants.^8^ This bushy shrub is widely distributed in dry and arid regions including Africa, Pakistan, India, Egypt, Jordan, Sudan, and Saudi Arabia.^9^

In Cholistan, Pakistan, the floral buds are utilized as vegetables and in pickles, while the leaves serve as cattle fodder. It has been reported that nearly all parts of the plant are used in traditional medicine practices, including Greco-Arab, Ayurveda, Chinese medicine, and Tibb-e-Unani. These parts are applied in the treatment of various ailments, such as arthritis, cholera, urinary purulent discharges, constipation, cough, intermittent fevers, puffiness, toothaches, asthma, dysentery, cardiac issues, soreness, and skin diseases.^8,10^ Several chemical and pharmacological researches have been carried out on *Capparis decidua* sterols,^11^ fatty acids,^12^ flavones,^13^ oxygenated heterocyclic constituents,^14^ alkaloids,^15,16^ and an isothiocyanate glucoside.^17^ These researches have been reported on different parts of this plant.

Water pollution is a pressing global issue, and numerous effective methods for wastewater treatment have been developed.^18^ Dyes, in particular, are extensively used in sectors such as textiles, footwear, leather, paper, food and beverages, personal care products, pharmaceuticals, and printing.^18,19^ The textile industry alone is responsible for the discharge of about 200 000 tonnes of dyes annually through its dyeing and finishing processes.^20,21^

Such large-scale effluents pose significant risks to human health and the environment by contaminating water sources, which reduces light penetration in aquatic ecosystems, adversely affecting marine life. Moreover, even at concentrations as low as 1 ppm, dyes released into the environment can be highly toxic and carcinogenic.

The photocatalytic degradation mechanism is an advanced method that has garnered significant attention for its potential to break the cycle of pollution by, for example, purifying wastewater, decomposing organic waste using light, or eliminating airborne pollutants. Recent advances in photocatalytic research have focused on developing efficient and reusable nanostructured materials for the degradation of various organic and inorganic pollutants. Several studies have demonstrated the versatility of metal oxide- and organic-based photocatalysts in removing dyes and pharmaceutical contaminants from water.^22–27^ Some studies indicate that the photocatalytic efficiency of ZnO for degrading different organic contaminants is comparable to that of the most effective TiO_2_ materials.^28,29^ Additionally, ZnO exhibits superior photocatalytic performance compared to TiO_2_ due to its higher quantum efficiency.^30^ ZnO has become one of the most extensively studied oxides, recognized as a favorable semiconductor for various applications, including wastewater treatment and biological research, owing to its high excitation energy, excellent photosensitivity, low toxicity, and affordability.^31,32^ Given its properties and findings, ZnO is a promising alternative to TiO_2_ in photocatalytic processes.^33,34^

Photocatalytic degradation of Rhodamine 6G (Rh 6G) continues to attract considerable research interest owing to its industrial prevalence and environmental persistence. Recent advances have focused on engineered nanocomposite photocatalysts that enhance visible-light absorption and promote efficient charge separation. For instance, a novel reduced graphene oxide–ZnO–Fe_2_O_3_ photocatalyst derived from zinc dross exhibited excellent visible-light-driven degradation of Rh 6G, achieving very high removal efficiencies.^35^ Furthermore, MgO NPs synthesized *via* precipitation methods demonstrated 93% Rh 6G removal under UV irradiation, underscoring the selective activity difference toward Rhodamine dyes.^36^ More recently, lead ferrite (PbFe_12_O_19_) NPs synthesized by sol–gel included magnetic separability and achieved >95% photocatalytic degradation of Rh 6G under optimized conditions.^37^

Herein, the biomimetic synthesis of ZnO NPs was conducted using *Capparis decidua* plant extract. *Capparis decidua* stem extract was selected as the biogenic chelating and stabilizing agent for ZnO NPs synthesis due to its rich content of bioactive phytochemicals such as flavonoids, alkaloids, and phenolics. These natural compounds act as efficient chelating and capping agents, facilitating the formation of stable and uniformly dispersed ZnO NPs without the need for toxic chemicals. Moreover, *Capparis decidua* is an easily available, eco-friendly, and medicinally important desert plant known for its strong antioxidant and antimicrobial properties, which further enhance the functional performance of the synthesized nanoparticles.

The photocatalytic activity of the synthesized ZnO NPs was systematically evaluated for the degradation of Rh 6G dye under various conditions. The results demonstrated exceptional photocatalytic efficiency, achieving a dye removal efficacy of 97% within a remarkably short duration of 75 min. Notably, this study represents the first reported instance of utilizing *Capparis decidua* plant extract for the eco-friendly synthesis of ZnO NPs, highlighting its potential as a sustainable alternative for nanomaterial fabrication. This innovative approach not only advances the field of green synthesis but also underscores the efficacy of biogenic ZnO NPs in environmental remediation applications.

The novelty of this work lies in the use of *Capparis decidua* stem extract—a plant not previously reported for ZnO synthesis—offering a new, sustainable phytochemical source. Additionally, we present a systematic investigation of how varying extract concentrations influence the structural, optical, and photocatalytic properties of ZnO NPs. This includes a clear correlation between extract concentration, particle size, and enhanced photocatalytic degradation efficiency. These aspects collectively underscore the uniqueness and value of our study.

## 2. Experimental

### 2.1. Materials

All reagents were of analytical grade. Zinc nitrate hexahydrate was purchased from Trading Dyamic Co. and Rhodamine 6G was purchased from Merck and were used as received.

### 2.2. Collection and preparation of plant specimens

Plant specimens were collected from their natural habitats, especially from a low basin at the Red Sea coastline south of Quseir, in the Eastern Desert includes a stretch of the Wadi Tundoub main channel as well as a deltaic plain. Latitudes 25°55′N, 34°11′E define its borders.

Plant material was collected in accordance with applicable national and international guidelines.^38^ Permission to collect the required plant species for scientific purposes was obtained from Botany and Microbiology department, Faculty of Science, Assiut University. According to Boulos (1999) the plant specimen was identified.

### 2.3. Preparation of plant extract

*Capparis decidua* stems were utilized in this study. Specimens were carefully washed using tap water and then distilled water to remove dust particles. Afterward, the specimens were air-dried and ground into a fine powder using a stainless-steel grinder. The plant powder was then preserved in airtight jars for subsequent use.

Three extracts were prepared at different concentrations, each extract was prepared by adding 2.0, 5.0 and 10.0 g of the dried powder of the plant to 200 mL of distilled water separately and heated at a temperature range of 50–60 °C for about 2 h. The obtained extract was cooled down to ambient temperature and finally sieved twice using Whatman filter paper. A translucent light yellowish extract with a pH of about 5 was obtained.

### 2.4. Synthesis of ZnO NPs

In a standard procedure, 8.0 g of zinc nitrate hexahydrate was mixed with 50 mL of *Capparis decidua* stems extract (three different concentrations were used separately). The mixture underwent a color change from a light yellowish hue to milky white upon dissolving the zinc salt, indicating the reaction and stabilization of the zinc compound. The mixture was heated at approximately 50 °C, and after about 2 h, the formation of the precipitates was completed. The precipitates were separated using a centrifuge (6000 rpm) then washed multiple times with dist. water, dried at 80 °C and annealed at 600 °C for 2 h.^39–41^ The prepared ZnO samples synthesized using varying extract concentrations were designated as 2Z, 5Z, and 10Z, corresponding to the respective concentrations. The schematic illustration for ZnO NPs synthesis is shown in Fig. S1.

### 2.5. Physical techniques

X-ray diffraction (XRD) analysis of the synthesized ZnO NPs was conducted using a Philips-type diffractometer (model 1700 with H.T.P.W 1730/104 KVA, utilizing Cu Kα radiation with a wavelength of 1.54180 Å). The XRD data was collected with the following parameters: a step size of 0.061, a duration of 0.60 seconds per step, a total acquisition time of 93.0 seconds, and a sample rotation speed of 15.0 rpm.

Fourier-transform infrared (FT-IR) spectroscopy of the prepared ZnO NPs was performed on a Shimadzu IR-470 spectrophotometer over the range of 4000–400 cm^−1^ using the KBr disc method. UV-visible spectroscopy of the nanoparticles suspended in water was conducted with a Shimadzu UV-2101 PC spectrophotometer, with the suspensions being sonicated for 30 min before measurement. UV-Vis spectrometer was utilized also for the determination of the Rh 6G initial and remaining concentrations after photocatalytic degradation process. To analyze the crystallinity, size, and morphology of the synthesized ZnO NPs, Hi Resolution Transmission Electron Microscope (JEOL JEM 2100, Japan) operating at 120 kV was utilized.

Nitrogen adsorption–desorption isotherms were obtained at −196 °C using a Quantachrome Instrument Corporation apparatus (Model Nova 3200). The samples were thoroughly degassed for 2 h at 150 °C before testing. With a JEOL (Germany) GmbH EDS X-ray photoelectron spectroscopic (XPS) analysis (model PHI 5000 Versaprobe) was conducted to study the chemical composition of the prepared ZnO samples. High-performance liquid chromatography (HPLC) was utilized to detect biocompounds in the ethanolic extract of *Capparis decidua* stem, employing an Agilent 1260 series system. Separation was conducted on an Eclipse C18 column (250 mm × 4.6 mm, 5 µm particle size), maintained at 40 °C. Each analysis involved the injection of a 5 µL sample. The mobile phase comprised water (solvent A) and 0.05% trifluoroacetic acid in acetonitrile (solvent B), delivered at a flow rate of 0.9 mL min^−1^ using a gradient elution. The gradient program was as follows: 0 min (82% A), 0–5 min (80% A), 5–8 min (60% A), 8–12 min (60% A), 12–15 min (82% A), 15–16 min (82% A), and 16–20 min (82% A). Detection was carried out at 280 nm using a multi-wavelength detector, and compounds were identified by matching their retention times with those of known phenolic and flavonoid standards.

### 2.6. Photocatalytic degradation

In a typical procedure, 50 mL of an aqueous Rh 6G solution with an initial concentration of 5.0 ppm was mixed with 50 mg of ZnO NPs as a heterogeneous photocatalyst. Before irradiation, the mixture was stirred for 30 min in the dark to reach an adsorption–desorption equilibrium. The mixture was then exposed to sunlight. At regular time intervals, 5.0 mL portions of the reaction mixture were taken, while the suspended photocatalyst was removed by centrifugation (6000 rpm). Ultimately, the residual Rh 6G concentration in the filtrate was determined using a UV-Vis spectrophotometer in the range 200 to 800 nm. Each photocatalytic test was conducted in three independent replicates, and the average values along with standard deviations were used for plotting.

## 3. Results and discussion

### 3.1. HPLC of *Capparis decidua* stem extract

HPLC analysis was performed to identify the major bioactive compounds present in the plant extract (Fig. S2). The chromatographic analysis identified gallic acid, chlorogenic acid, and catechin as the major constituents, their structures are illustrated in Fig. S3. These compounds share common structural features characteristic of phenolic molecules, including hydroxyl groups attached to aromatic rings. In particular, the presence of carboxylic acid groups in gallic and chlorogenic acids suggests their potential for strong hydrogen bonding and metal-chelating activity. These phytochemicals are well-known for their strong antioxidant and chelating properties, which may play a crucial role in the biosynthesis and stabilization of nanoparticles.

### 3.2. Role of plant extract in ZnO NPs formation

As indicated by the phytochemical profiling of the *Capparis decidua* stem extract gallic acid, chlorogenic acid, and catechin are the major constituents. These constituents play synergistic roles in the green synthesis of ZnO NPs. Although zinc remains in the +2 oxidation state in ZnO, these polyphenolic compounds significantly influence the formation mechanism through complexation, hydrolysis, and stabilization processes. Gallic acid, a well-known polyphenol with multiple hydroxyl and carboxyl groups, readily coordinates with Zn^2+^ ions, facilitating the controlled hydrolysis of zinc species into Zn–OH intermediates. During the subsequent condensation *via* dehydration, it binds to the ZnO NPs surface, acting as a capping and stabilizing agent that influences particle morphology.^42,43^ Similarly, chlorogenic acid—an ester of caffeic and quinic acids—exhibits both antioxidant and metal-chelating properties, enabling it to regulate the nucleation and growth of ZnO NPs while preventing agglomeration through surface adsorption.^44^ Catechin, a flavonoid rich in hydroxyl groups, further contributes by forming strong hydrogen-bonding and electrostatic interactions with the ZnO surface, thereby improving nanoparticle dispersion, stability, and biocompatibility.^44,45^ Collectively, these phytochemicals act as coordinating, capping, and structure-directing agents, ensuring an eco-friendly synthesis route that yields uniformly dispersed and stable ZnO NPs.^46,47^

### 3.3. Mechanism of ZnO NPs formation

The green synthesis of ZnO NPs using *Capparis decidua* stem extract proceeds through a coordination-hydrolysis-nucleation-growth-stabilization mechanism, primarily mediated by the bioactive phytochemicals present in the extract.^47^ Initially, zinc precursor (Zn^2+^ ions) in the aqueous solution interacts with the polyphenolic compounds in the extract, mainly gallic acid, chlorogenic acid, and catechin. These molecules possess multiple hydroxyl groups capable of chelating Zn^2+^ ions, which leads to the hydrolysis of Zn^2+^ to Zn(OH)_2_, followed by thermal decomposition into ZnO nuclei during the calcination process. Once nucleation begins, the phytochemicals serve as capping and stabilizing agents, adsorbing onto the surface of the ZnO nuclei. This surface adsorption prevents excessive particle aggregation, regulates the growth of the particles, and helps control the morphology and size distribution. The simplified reaction pathway can be summarized as:

1 Zn^2+^ + OH^−^ → Zn(OH)_2_ → ZnO (upon heating)

The green synthesis mechanism is supported by several recent studies reporting the use of plant extracts rich in polyphenols and flavonoids for ZnO NPs preparation.^44,45^ The formation mechanism, supported by FTIR data discussed in Section 3.5. The FT-IR spectra provide evidence for the involvement of plant-derived functional groups during the synthesis process. In the extract, characteristic peaks corresponding to –OH (broad band around 3400 cm^−1^), C=O (around 1700 cm^−1^), and C–O (around 1200–1300 cm^−1^) are clearly observed, indicating the presence of phenolic and carboxylic compounds such as gallic acid, chlorogenic acid, and catechin.

### 3.4. XRD analysis of the samples

**Fig. 1:**
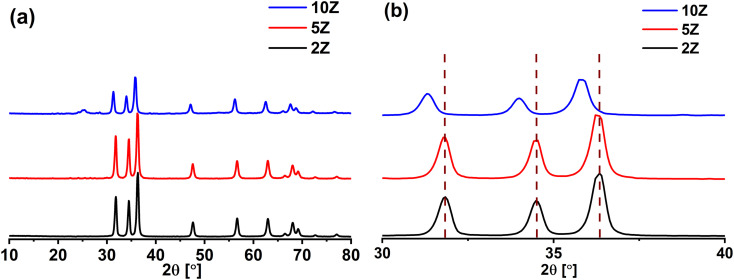
(a) XRD patterns of ZnO NPs prepared using different plant extract concentrations, and (b) the corresponding magnified XRD patterns.

X-ray diffraction (XRD) was employed to assess the crystallinity, phase purity, and structure of ZnO NPs synthesized using varying concentrations of *Capparis decidua* stem extract. The findings offer valuable insights into how the concentration of the plant extract affects the crystallinity and phase composition of the produced ZnO NPs. The XRD patterns (Fig. 1a) for all ZnO samples display distinct diffraction peaks that align with the hexagonal wurtzite structure of ZnO, as referenced by the JCPDS card no. 36-1451. The prominent peaks observed at 2*θ* values of approximately 31.8°, 34.4°, 36.3°, 47.5°, 56.6°, 62.9°, and 68.0° can be indexed to the (100), (002), (101), (102), (110), (103), and (112) crystal planes, respectively.^48,49^ This confirms the successful synthesis of ZnO NPs with high phase purity, particularly for the samples 2Z and 5Z. For the sample 10Z a tiny peak is apparent at 2*θ* of ∼25°, that could be related to the presence of carbonaceous material incorporated with ZnO NPs. For the other two ZnO NPs samples the carbonaceous content cannot be detected *via* XRD analysis and further analysis is needed to establish its presence.

The XRD patterns reveal that ZnO NPs prepared from the highest plant extract concentration (10Z) display broader and less intense peaks, indicating lower crystallinity and smaller crystallite size. In contrast, ZnO NPs obtained from the lowest extract concentration (2Z) show sharper, more intense peaks, reflecting better crystallinity and larger crystallite size.

There are number of reasons behind this phenomenon. A higher concentration of plant extract may lead to a higher density of organic molecules (*e.g.*, polyphenols, flavonoids) that cap the nanoparticles during the synthesis. This capping effect can restrict the growth of crystallites, resulting in crystallites with smaller sizes and less ordered structures. Additionally, the organic residues from the plant extract may interfere with the crystallization process during heat treatment, leading to broader XRD peaks. On contrast, a lower concentration of plant extract provides fewer capping agents, allowing the nanoparticles to grow more freely during synthesis and heat treatment. This results in larger crystallites and a more ordered crystalline structure, which is reflected by the sharper XRD peaks. The crystallite size of the ZnO NPs was determined using the Debye–Scherrer equation:2
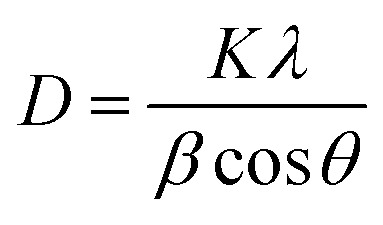
where *D* is the crystallite size, *K* is the Scherrer constant (0.9), *λ* is the X-ray wavelength (1.5406 Å), *β* is the full width at half maximum (FWHM) of the diffraction peak, and *θ* is the Bragg angle.

The ZnO NPs prepared with the highest plant extract concentration (10Z) exhibited an average crystallite size of 17.9 nm, compared to 20 nm for those obtained from the lowest extract concentration (2Z). This trend aligns with the observed peak broadening and sharpening in the XRD patterns.

#### 3.4.1. Influence of plant extract on crystal growth

The plant extract plays a dual role as a capping and a stabilizing agent during the synthesis of ZnO NPs. The concentration of the plant extract directly influences the kinetics of nucleation and crystal growth:

• A lower concentration of plant extract likely reduces the number of active biomolecules or functional groups available to act as nucleation sites. With fewer nucleation sites, the crystallites grow larger during the synthesis process, leading to sharper and more intense XRD peaks that indicate larger crystallite sizes and improved crystallinity.

• A reduced concentration of plant extract may result in a more controlled environment for crystal growth. Excessive biomolecules from the extract can interfere with crystal growth, causing defects or smaller crystallites. The lower concentration minimizes this interference, allowing the crystals to grow more uniformly and achieve better crystallinity.

• Plant extracts often contain capping agents like flavonoids, phenols, or alkaloids, which can limit crystal growth by adsorbing onto the surface of formed particles. At lower extract concentrations, the capping agents are less abundant, allowing the crystals to grow larger and develop a more ordered structure.

• High concentrations of plant extract can accelerate reaction kinetics, leading to the rapid formation of smaller, less crystalline particles. A lower concentration slows the reaction, providing sufficient time for the formation of larger and well-ordered crystals.

• Higher concentrations of plant extract can lead to the incorporation of more organic material or amorphous phases in the product, which can weaken the XRD peak intensity and broadening (as shown by XRD pattern of sample 10Z). The lower concentration of plant extract reduces the amorphous content, improving the sharpness and intensity of XRD peaks.

#### 3.4.2. Influence of plant extract on crystal structure

Interestingly, a shift in XRD peak positions to lower 2*θ* angles with increasing plant extract concentration is obvious (Fig. 1b) that could be attributed to changes in the lattice parameters or strain within the crystal structure of the synthesized nanoparticles.

According to Bragg's law (*nλ* = 2*d* sin *θ*), the diffraction peak position (2*θ*) is inversely related to the interplanar spacing (*d*) of the crystal lattice. A shift to lower 2*θ* angles indicates an increase in the interplanar spacing (*d*). Possible causes of increased interplanar spacing are lattice expansion, strain effects or defect formation. In our case, the peak shift can be attributed to microstructural and surface effects rather than true lattice expansion. Higher extract concentrations reduce crystallite size, causing peak broadening and overlap that can make peaks appear at lower angles. Additionally, organic molecules from the extract can induce local strain and defects within the ZnO lattice and slightly distort near-surface planes, affecting peak positions.

#### 3.4.3. Rietveld analysis

Structural analysis of the ZnO samples was conducted using the Rietveld refinement method (Fig. S4) to investigate the influence of preparation methods, specifically the concentration of plant extract, on the structural properties of the samples. The refinement data for the three ZnO samples are summarized in Table 1.

**Table 1:** Measurement parameters, structure and refinement data of the different ZnO samples

	2Z	5Z	10Z
Formula sum	Zn_2.00_O_2.00_	Zn_2.00_O_2.00_	Zn_2.00_O_2.00_
Formula mass, g mol^−1^	162.76	162.76	162.76
Density (calcd), g cm^−3^	5.68	5.69	5.70
*a* = *b*, Å	3.2486(2)	3.2481(2)	3.2445(6)
*c*, Å	5.2020(3)	5.2008(4)	5.196(1)
*c*/*a* ratio	1.6013	1.6012	1.6015
*β*, °	120	120	120
*V*, 10^6^ pm^3^	47.543	47.518	47.371
*R* (expected), %	6.697	6.434	6.679
*R* (profile), %	5.996	6.552	10.661
*R* (weighted profile), %	7.846	8.735	17.645
*R* (bragg), %	2.507	2.826	2.351
GOF	1.373	1.843	6.978

The results reveal as the concentration of the plant extract increases, the unit cell volume of the ZnO structure decreases. This is accompanied by a noticeable contraction in the lattice parameters along both the “*a*” and “*c*” axes, with the most significant reduction observed in the sample synthesized using the highest concentration of plant extract. This phenomenon suggests that the incorporation of higher concentrations of plant extract during synthesis induces structural compression, likely due to changes in the nucleation and growth dynamics of the ZnO crystals. Notably, the *c*/*a* ratio for all samples remains approximately 1.6, which is in close agreement with the ideal value of 1.633 for a hexagonal wurtzite structure. This consistency indicates that the hexagonal symmetry of the ZnO lattice is preserved across all samples, despite the observed variations in unit cell dimensions. The findings highlight the critical role of synthesis conditions, particularly the concentration of plant-derived additives, in modulating the structural properties of ZnO, which may have implications for tailoring its functional characteristics in various applications.

### 3.5. FTIR analysis of the samples

**Fig. 2:**
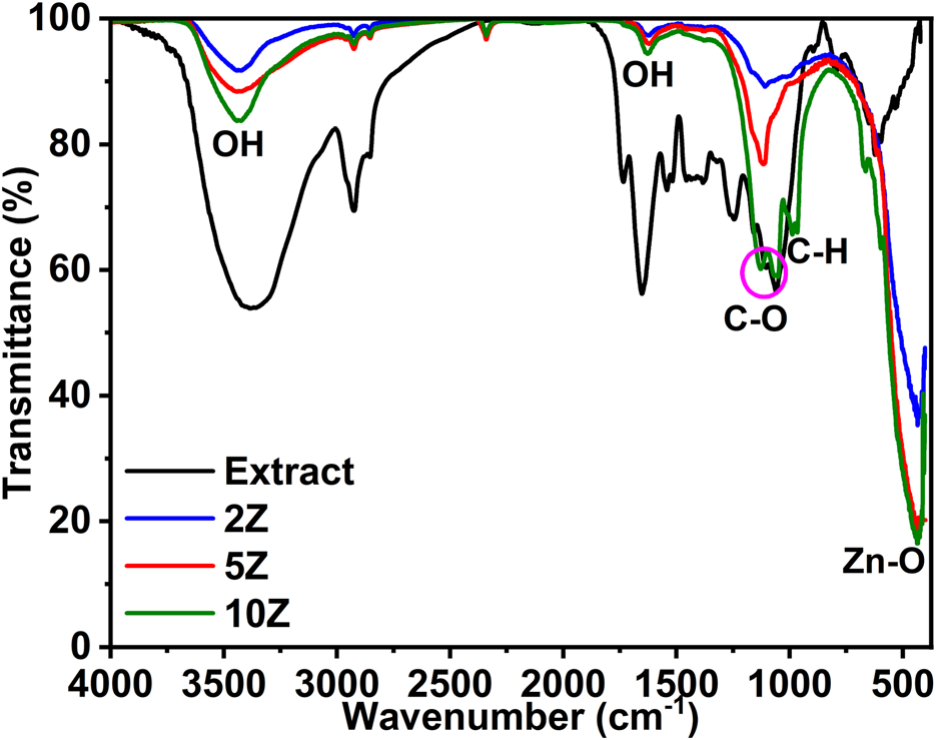
FTIR spectra of the plant extract and ZnO NPs prepared using different extract concentrations.

FTIR analysis was conducted for the plant extract and the ZnO samples synthesized with varying extract concentrations (Fig. 2). From the spectra we get information regarding the chemical bonding between Zn and O atoms in addition to the played role of the plant extract in ZnO formation. The FTIR spectra in Fig. 2 show a distinct absorption band at 428 cm^−1^, corresponding to Zn–O stretching vibrations, confirming the formation of ZnO in the synthesized material.^48,49^ This band is slightly shifted from that of chemically prepared ZnO (434 cm^−1^). The phytochemicals identified in the plant extract—mainly gallic acid, chlorogenic acid, and catechin—induced additional FTIR absorption bands at 970–1150 cm^−1^. These bands corresponding to C–H out-of-plane bending, C–O stretching, and other carbon–oxygen vibrations of residual organic species. Notably, these peaks were relatively weak in the ZnO sample synthesized with the lowest extract concentration (2Z), suggesting minimal phytochemical capping and limited organic interaction at the nanoparticle surface. In contrast, the intensity of these bands increased significantly with higher extract concentrations, as observed in sample 10Z, indicating a greater abundance of phytochemicals involved in surface stabilization and capping.

Additionally, two prominent absorption bands appear at 3453 and 1653 cm^−1^, which can be attributed to the O–H stretching and bending vibrations, respectively. Notably, the intensity of these bands increases as the concentration of the plant extract rises. This enhancement in band intensity may be linked to a higher presence of surface hydroxyl groups, which in turn suggests an increased contribution of organic species from the extract. Moreover, the variation in intensity may also be correlated with changes in particle size among the synthesized samples. Higher extract concentrations can influence nucleation and growth processes during synthesis, leading to smaller particles with higher surface area and, consequently, more exposed functional groups. This trend supports the role of plant-derived compounds in modulating nanoparticle surface chemistry, potentially enhancing the stability, dispersibility, and functionality of the ZnO nanoparticles through more effective surface passivation.

### 3.6. Surface properties of ZnO NPs

The surface properties of ZnO NPs synthesized using different plant extract concentration *i.e.* 2Z, 5Z and 10Z was explored employing the nitrogen adsorption–desorption technique. By applying the BET approach, the adsorption–desorption isotherms were received and plotted as shown in Fig. S5. For all samples, the obtained isotherms were classified as type IV, featured with a hysteresis which suggests the presence of mesopores. In this class, the capillary condensation linked to the mesopores restricts the adsorption at high *P*/*P*°. The BET surface area analysis revealed a clear correlation between concentration of the plant extract used during synthesis and the surface area of the resulting ZnO NPs. The lowest surface area (2.2 m^2^ g^−1^) was observed for the sample synthesized with the minimal extract concentration (2Z), suggesting limited organic interaction and minimal structural modification. As the extract concentration increased, the surface area of ZnO samples also increased, reaching a maximum of 32.9 m^2^ g^−1^ for the 10Z sample. This trend can be attributed to the higher content of carbonaceous material left from the extract's phytochemicals (evidence by FTIR and XRD analyses), which results in a more porous structure. On the other hand, the samples show mesoporous structure, as demonstrated by their average pore diameters of 20.2, 19.6, and 17.2 nm. Pore volume was also measured, and the sample that was obtained with the highest concentration of plant extract had the biggest pore volume. It may have the largest specific surface area for this reason. The detailed physicochemical characteristics of ZnO NPs produced with varying concentration of the plant extract are shown in Table 2.

**Table 2:** Surface area, average pore width and total pore volume for ZnO samples prepared using different plant extract concentrations

	2Z	5Z	10Z
*S*_BET_ (m^2^ g^−1^)	2.2	6.7	32.9
Pore volume (cm^3^ g^−1^)	0.008	0.011	0.039
Pore width (nm)	20.2	19.6	17.2

### 3.7. XPS analysis

**Fig. 3:**
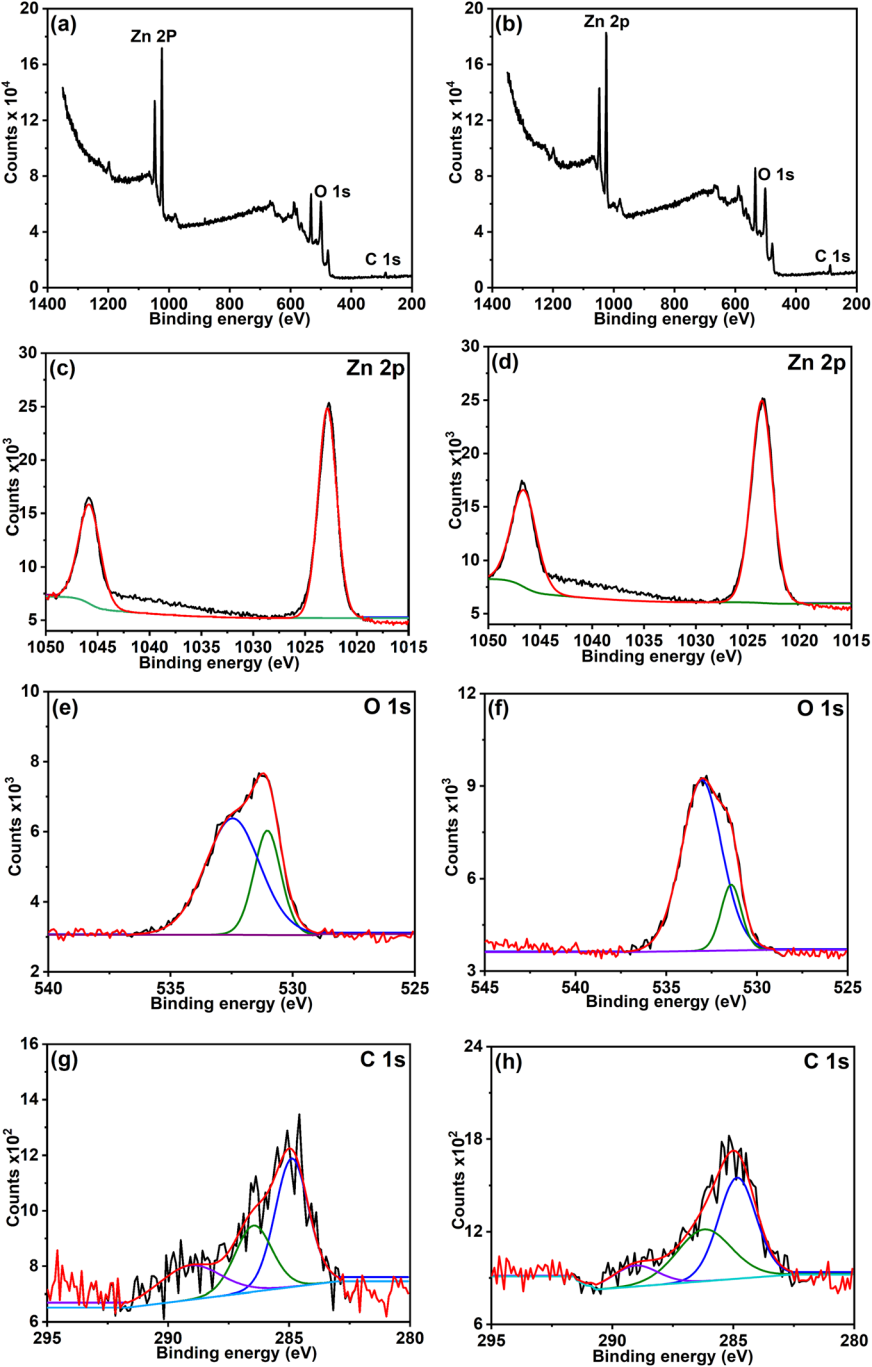
XPS analysis of ZnO NPs samples 2Z and 10Z, respectively. (a and b) Survey spectra, and high-resolution spectra of (c and d) Zn 2p, (e and f) O 1s, and (g and h) C 1s.

X-ray photoelectron spectroscopy (XPS) analysis was performed to investigate the surface composition and chemical states of ZnO samples synthesized using lowest (2Z) and highest (10Z) concentrations of the plant extract. The wide scan analysis showed the presence of Zn, O and C elements in the samples (Fig. 3). Both samples exhibited characteristic Zn 2p_3/2_ and Zn 2p_1/2_ peaks at ∼1023.5 eV and ∼1046.6 eV, respectively, confirming the presence of Zn^2+^ in the wurtzite ZnO structure. Slight positive shifts in these binding energies compared to conventionally synthesized ZnO (1021.5 and 1044.7 eV) were observed,^50^ which can be attributed to surface interactions between ZnO and oxygen-containing functional groups of carbonaceous material left from the phytochemicals in the plant extract. The 10Z sample showed slightly reduced Zn 2p peak intensities, likely due to the higher surface coverage of organic matter derived from the abundant phytochemicals in the plant extract.

The O 1s spectra for both samples revealed a dominant peak around 531.1 eV corresponding to lattice oxygen, along with a secondary peak at 532.4 eV, attributed to surface hydroxyl groups and adsorbed oxygen species. Notably, the 10Z sample exhibited a higher relative intensity of this secondary component, indicating an increased presence of surface-bound oxygen-containing functionalities. The C 1s spectrum further supported this observation, as the 10Z sample showed more intense peaks at ∼286.2 eV and ∼288.9 eV, corresponding to C–O and O–C=O groups, in addition to the main C–C/C–H peak at ∼284.8 eV. These findings demonstrate that increasing the plant extract concentration during synthesis leads to a greater incorporation of carbonaceous material on the ZnO surface, enhancing the material's surface functionalization and potentially influencing its physicochemical properties.

### 3.8. Morphological investigation

**Fig. 4:**
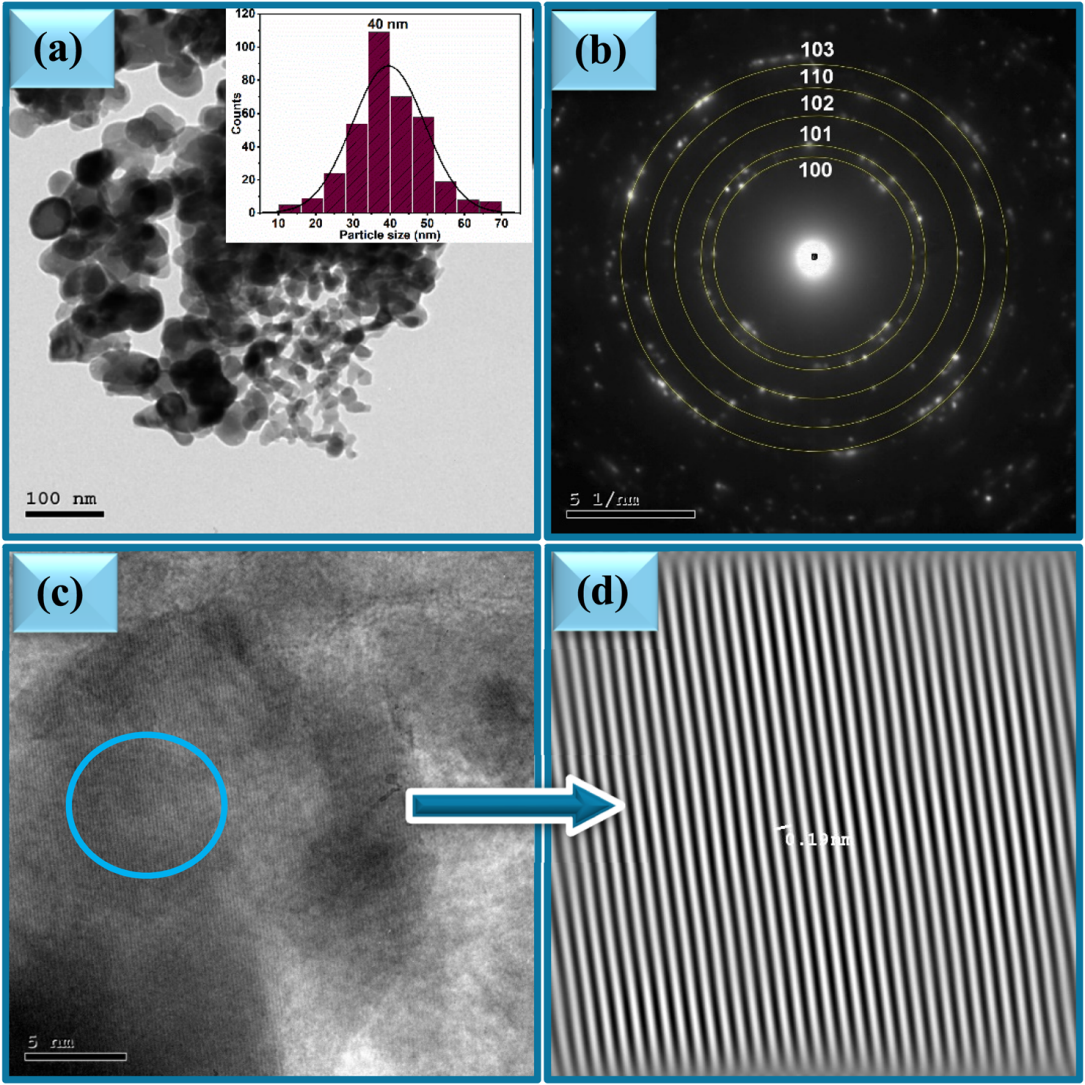
(a) TEM, (b) SAED and (c and d) HR-TEM of ZnO NPs, sample 2Z.

**Fig. 5:**
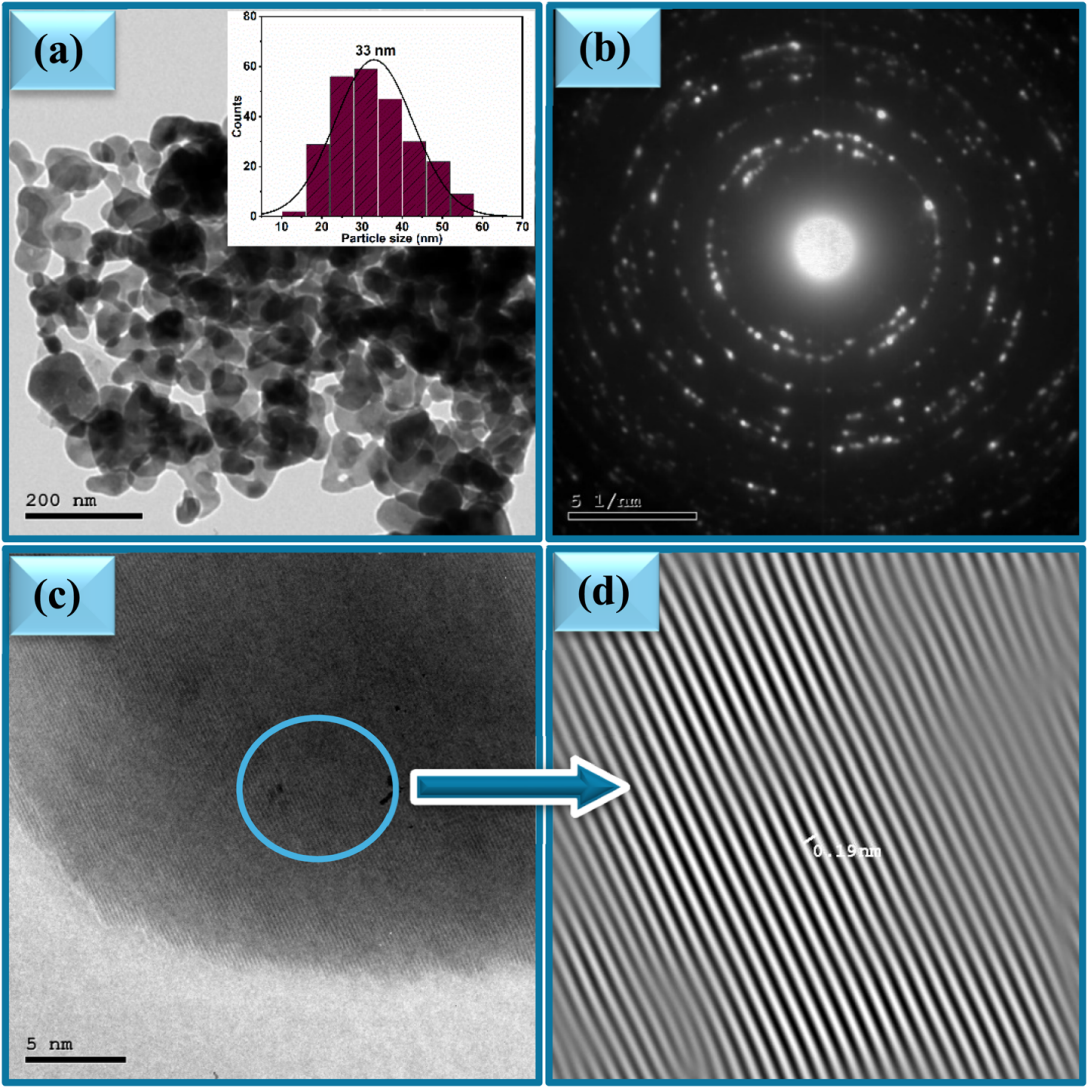
(a) TEM, (b) SAED and (c and d) HR-TEM of ZnO NPs, sample 5Z.

**Fig. 6:**
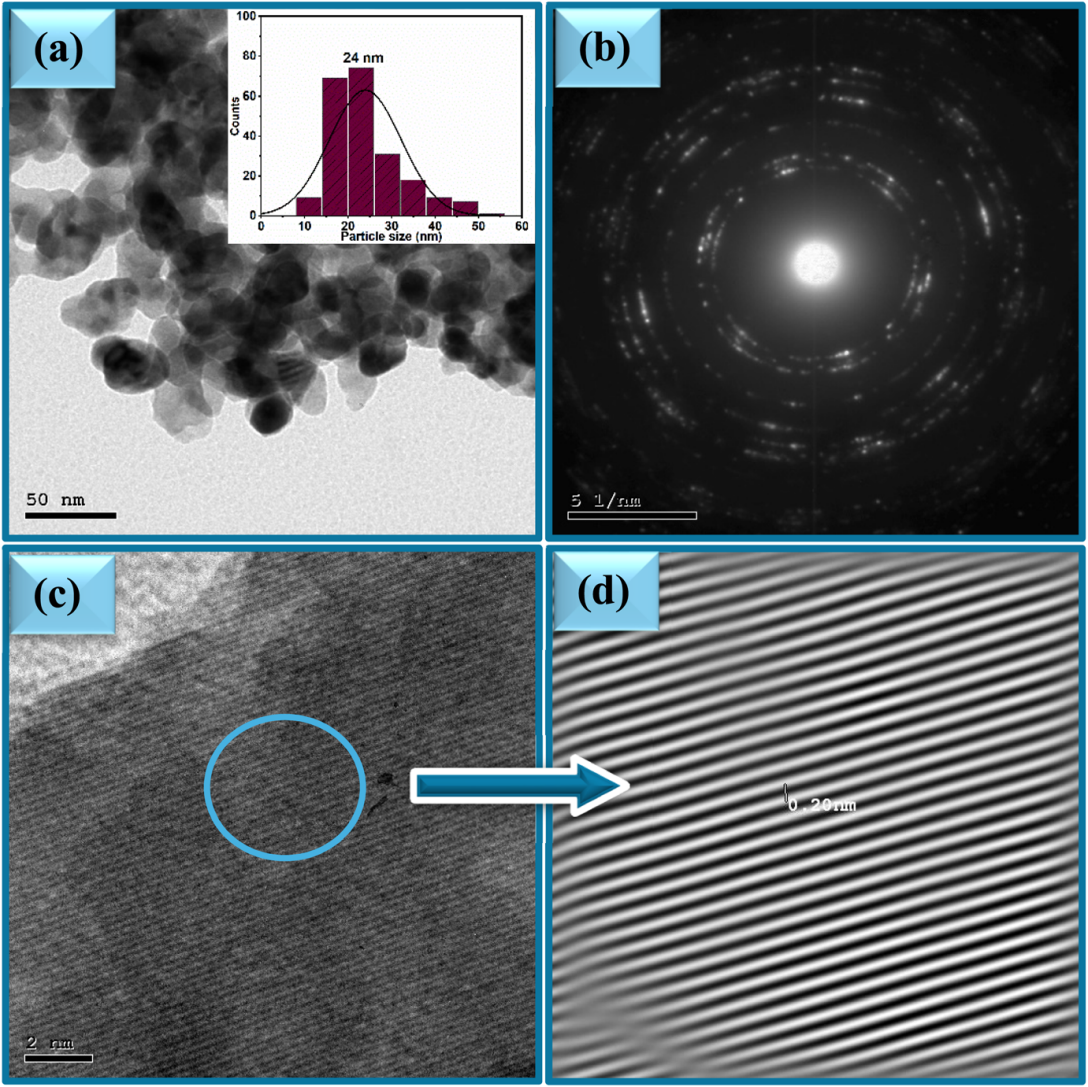
(a) TEM, (b) SAED and (c and d) HR-TEM of ZnO NPs, sample 10Z.

The effect of varying the plant extract concentrations on the morphology of the synthesized ZnO NPs was systematically investigated using Transmission Electron Microscopy (TEM). TEM images of the samples 2Z, 5Z, and 10Z are presented in Fig. 4a, 5a and 6a. All samples exhibited predominantly spherical morphologies. Notably, the 2Z sample—synthesized using the lowest concentration of plant extract—displayed the largest particle size, with an average diameter of approximately 40 nm and a broad size distribution ranging from 10 to 70 nm. Conversely, the 10Z sample, which was prepared using the highest extract concentration, demonstrated the smallest particle size, averaging around 24 nm with a narrower size distribution (10–50 nm). The 5Z sample exhibited an intermediate average particle size of 33 nm and a moderate size distribution between 10 and 57 nm. These morphological trends are consistent with the XRD findings, which similarly indicated a decrease in the crystallite size with increasing concentrations of the plant extract. The TEM analysis clearly illustrates a correlation between the plant extract concentration and the resulting particle size and distribution size of ZnO NPs. Higher concentrations of the extract appear to provide a greater abundance of bioactive molecules, which can act as stabilizing and capping agents. This facilitates better control over nucleation and growth processes during nanoparticle formation, thereby yielding smaller and more uniformly sized particles. The 10Z sample, prepared with the highest extract concentration, showed the narrowest size distribution and smallest average diameter, suggesting enhanced stabilization during synthesis. In contrast, the 2Z sample, with minimal plant extract, exhibited less control over growth, leading to broader and larger particle distributions. This size trend aligns well with XRD data, reinforcing the conclusion that increasing the bioavailability of phytochemicals in the extract promotes the formation of smaller ZnO crystallites with more uniform morphology. These findings highlight the importance of extract concentration as a key parameter in tuning nanoparticle properties for targeted applications. SAED patterns of ZnO samples are shown in Fig. 4b, 5b and 6b. The ring patterns are indexed which approved the phase purity of the ZnO nanospheres as depicted by XRD results. Additionally, it signified the polycrystalline nature of the prepared nanoparticles. Furthermore, HRTEM images of the ZnO nanospheres shown in Fig. 4c, 5c and 6c indicated also the crystalline nature. It had a uniform structure with a periodic fringe spacing of 0.19 nm (Fig. 4d, 5d and 6d) which corresponds to the interplanar spacing between the (102) planes of the hexagonal ZnO structure.

### 3.9. Optical properties

**Fig. 7:**
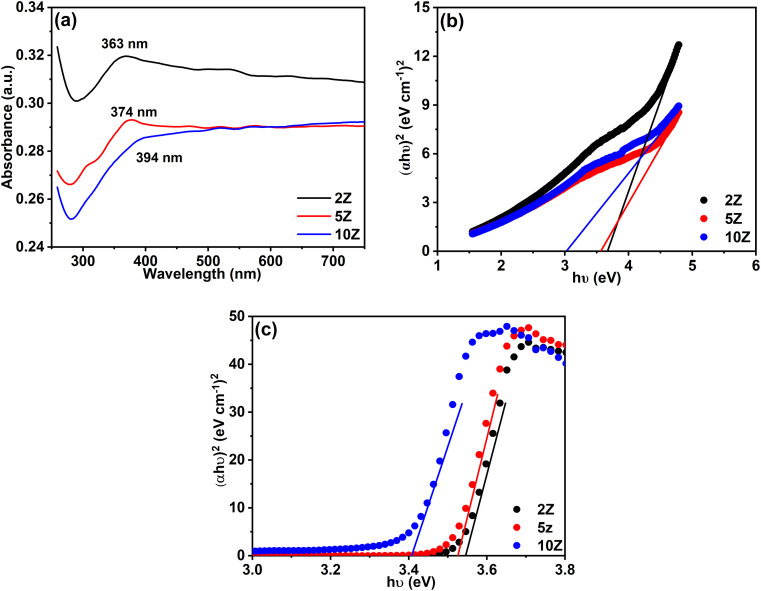
(a) UV-Vis spectra of ZnO samples suspended in water, (b) the corresponding Tauc's plots, and (c) Tauc's plots derived from UV-Vis diffuse reflectance spectra.

The optical properties of the three ZnO samples prepared using different plant extract concentrations were investigated using UV-Vis spectroscopy. The absorption spectra of ZnO samples dispersed in water (Fig. 7a) show broad absorption peaks revealing their high absorption character. For the sample obtained using the lowest plant extract concentration (2Z), an absorption peak is apparent at *λ*_max_ = 363 nm that is related to the electronic transition from the valence to the conduction band. This band is a characteristic band for ZnO. Red shift in the peak position (*i.e.* 363, 374, and 394 nm) occurs with increasing plant extract concentration.

The optical band gaps were derived through analysis of the absorption spectra. Tauc's model for direct allowed inter-band optical transitions was applied, which is expressed by the following equation:

3 (*αhν*)^2^ = *β*(*hν* − *E*^opt^_g_)

The parameter *α* represents the absorption coefficient, *hν* denotes the photon energy, *E*_g_ corresponds to the optical band gap, and *β* signifies the steepness parameter, which is indicative of the degree of structural order within the material.

Fig. 7b presents Tauc's plots, depicting the relationship between (*αhν*)^2^ and *hν* for the three ZnO samples. The optical bandgap energy (*E*_g_) was determined from the extrapolation of the linear region of the plots. Notably, the sample 10Z, synthesized using the highest concentration of the plant extract, exhibited the lowest bandgap energy of 3.02 eV. In contrast, the sample 2Z, prepared with the lowest plant extract concentration, demonstrated the highest bandgap of 3.67 eV. Moreover, the sample 5Z, fabricated with an intermediate plant extract concentration, displayed an intermediate bandgap energy of 3.55 eV. This variation in the bandgap energies can be attributed to the influence of plant extract concentration on the structural and electronic properties of the ZnO samples. The observed ZnO bandgap values are consistent with findings reported in several previous studies.^51^ Notably, the optical bandgap energies of the synthesized ZnO NPs exhibited a clear dependence on the concentration of plant extract used during the synthesis.

An unexpected relationship between particle size and bandgap energy is observed in the ZnO samples, which is more likely attributed to surface-related effects rather than pure quantum confinement, as the particle sizes remain above the ZnO Bohr exciton radius. Sample 10Z, synthesized with the highest plant extract concentration, exhibited the smallest particle size and lowest bandgap (3.02 eV), which can be attributed to increased surface defects and oxygen vacancies.^52^ These factors, along with the strong surface interaction with carbonaceous species from the plant extract, introduce localized energy states near the conduction or valence bands. These additional states effectively narrow the optical bandgap by facilitating electronic transitions at lower energies. Moreover, enhanced surface defects and lattice strain in smaller particles can further contribute to band structure modification, resulting in the reduced bandgap value. In contrast, sample 2Z, prepared with the lowest extract concentration, showed the largest particle size and highest bandgap (3.67 eV), reflecting fewer surface defects and better crystal quality. The intermediate properties of sample 5Z (bandgap 3.55 eV) further support this trend, highlighting the role of plant extract concentration in modulating particle size and electronic structure during the green synthesis.

On the other hand, the band gap energies of the ZnO samples were evaluated based on UV-Vis diffuse reflectance spectra (not shown), and the corresponding results are presented in Fig. 7c. A similar trend was observed, where the 2Z sample exhibited the highest band gap energy (3.56 eV), while the 10Z sample showed the lowest value (3.33 eV). The variations in band gap values could be attributed to differences in the methods used for recording the UV-Vis spectra.

### 3.10. Photocatalytic removal of Rh 6G

Prior to the study of the photocatalytic activity of the synthesized ZnO NPs (sample 2Z), a blank solution of the Rhodamine 6G (Rh 6G) dye was subjected to sunlight in the absence of the photocatalyst. As illustrated in Fig. S6a, the irradiation of the dye over 45 min results in a slight change of its concentration (12.3% removal). The removal efficiency (*R*%) of the Rh 6G was estimated using the following relationship:4
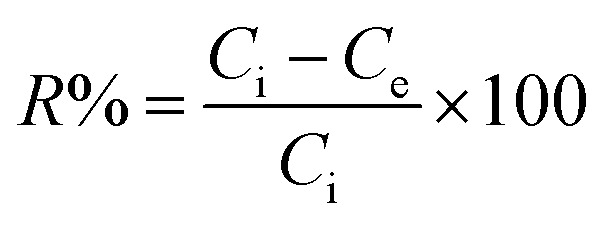
where *C*_i_ and *C*_e_ (mg L^−1^) are the initial and the equilibrium concentrations of the dye solution, respectively.

The amount adsorbed of organic pollutants on nanoparticles surface has a significant impact on their photocatalytic efficiency. Dark part experiment was performed for 45 min to explore the adsorption level of Rh 6G upon ZnO surface. The results revealed a little change in the Rh 6G concentration that may occur due to the adsorption process (Fig. S6b).

**Fig. 8:**
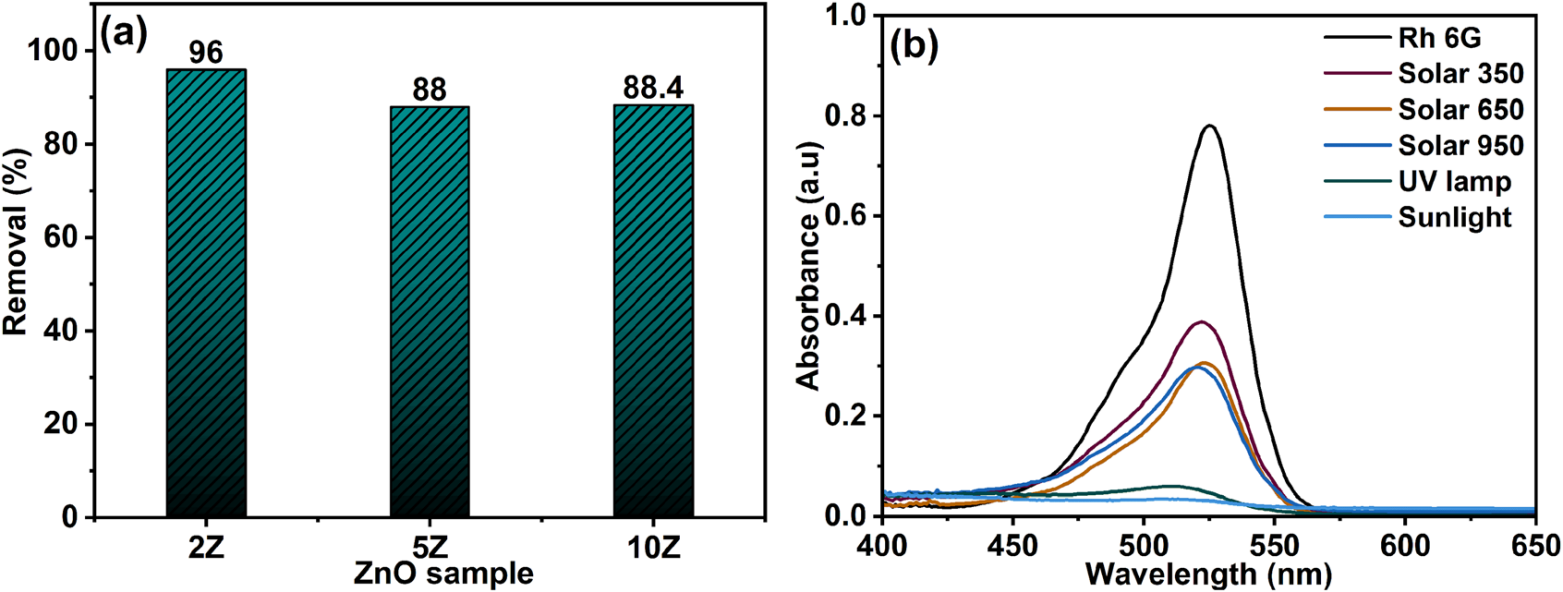
(a) Removal of 50 mL (5.0 ppm) Rh 6G using 50 mg of various ZnO samples and (b) effect of various light sources on the degradation of Rh 6G.

The photocatalytic efficiency of various ZnO samples for the degradation of Rh 6G was systematically evaluated. The results revealed that sample 2Z exhibited superior photocatalytic performance, achieving a degradation efficiency of 96%, significantly higher than the efficiencies of 88% and 88.4% observed for the other two samples (5Z and 10Z respectively, as shown in Fig. 8a). Consequently, sample 2Z was selected for all subsequent photocatalytic experiments due to its enhanced activity.

Despite the large surface area of 10Z, this sample did not demonstrate the highest photocatalytic efficiency. Instead, the 2Z sample achieved the most effective degradation of Rh 6G, with a degradation efficiency of 96%, compared to ∼88% for both 5Z and 10Z. This counterintuitive result suggests that surface area alone is not the sole determinant of photocatalytic activity. One possible explanation is that the 2Z sample exhibits the widest bandgap (3.67 eV), which may enhance the generation of reactive oxygen species under UV excitation, thereby increasing the photocatalytic activity. In contrast, the narrower bandgap of 10Z (3.02 eV) may have facilitated faster electron–hole recombination, limiting the number of reactive species available for dye degradation. Furthermore, the excessive presence of plant-derived organics in 10Z could have introduced surface defects or residual carbonaceous layers that hinder light absorption or block active catalytic sites, thereby reducing photocatalytic efficiency despite the high surface area.

Another explanation for the higher photocatalytic performance of the 2Z sample could be attributed to its relatively lower defect density and reduced surface recombination sites rather than its wider bandgap. Although 2Z possesses the highest bandgap energy, its larger crystallite size and lower concentration of oxygen vacancies contribute to more efficient charge separation and longer carrier lifetimes. Consequently, fewer defect-induced recombination centers enhance the generation and participation of photogenerated electrons and holes in redox reactions, improving the overall photocatalytic efficiency.

On the other hand, the influence of different light sources on the photocatalytic performance was examined using a solar simulator (at various light intensities of 350, 650 and 950 W m^−2^) and a UV lamp (350 nm). As illustrated in Fig. 8b, the highest degradation efficiency of Rh 6G was achieved under sunlight irradiation (∼96%), followed by UV light (∼94%). In contrast, the solar simulator produced low degradation efficiency (49.7%), even with increasing light intensity. The degradation rate increased up to a certain threshold reaching 60.1% and then plateaued, indicating a saturation effect.

Furthermore Table 3 presents a comparison of the degradation efficiency of the green-synthesized ZnO NPs (2Z) with those prepared by conventional chemical methods. Notably, the 2Z sample demonstrates superior degradation efficiency and a shorter reaction time compared to its chemically synthesized counterparts.

**Table 3:** Comparison of the degradation efficiency of ZnO NPs (sample 2Z) with those of ZnO NPs prepared chemically

No.	Synthesis methods	Dye concentration	Source of irradiation	Time of removal	Removal (%)	Ref.
(1)	Sol–gel	10, 20, and 30 ppm	Visible lamp	150 min	53.7, 20.85, and 11.0	53
(2)	Polyol	2.5 × 10^−5^ M	UV light	180 min	68.68	54
(3)	Sol–gel	10 ppm	Visible light	240 min	37.0 ± 2.9	35
(4)	Green	5 ppm	Sunlight	75 min	96	Current study

The dye degradation process was evaluated based on three critical parameters: photocatalyst concentration, pH of the reaction medium, and initial dye concentration. To systematically assess their individual and combined effects on the process, a controlled experimental approach was employed. Specifically, two of the three parameters were maintained at constant values, while the third was systematically varied. This methodology allowed for the isolation and analysis of the influence of each parameter on the degradation efficiency.

#### 3.10.1. The influence of initial dye concentration

**Fig. 9:**
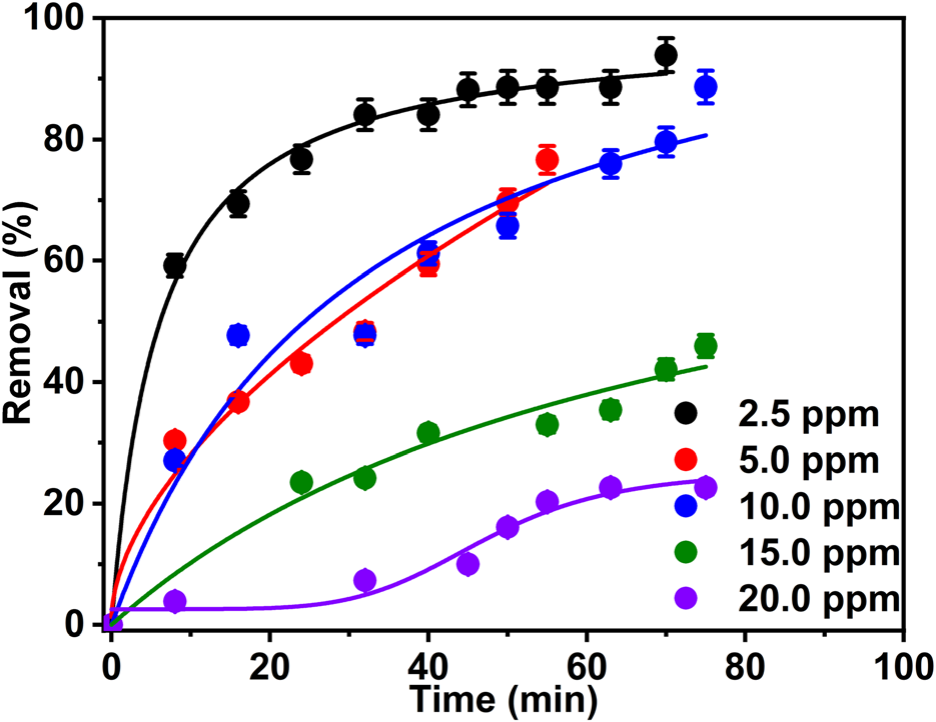
Effect of initial Rh 6G concentration on the degradation efficiency of 50 mL dye solution using 50 mg of ZnO NPs (sample 2Z) as photocatalyst.

To investigate the effect of dye concentration on the degradation process, the experiments were conducted under controlled conditions, with the pH maintained at 6.5 and the catalyst mass (sample 2Z) fixed at 50 mg. Initial dye concentrations of 2.5, 5.0, 10.0, 15.0, and 20.0 ppm were tested. The UV-Vis spectra, shown in Fig. S7a–e, exhibited the degradation of Rh 6G across the different initial Rh 6G concentrations. The results demonstrate a significant improvement in the removal efficiency of Rh 6G at lower dye concentrations (2.5, 5.0, and 10.0 ppm). Specifically, at 2.5 ppm, degradation of 94% of the Rh 6G dye was achieved after 46 min of sunlight exposure (Fig. 9). When using 5.0 and 10.0 ppm as initial dye concentrations, degradation efficiencies reached 96% and 89%, respectively, however after 75 min of sunlight exposure (Fig. 9).

In contrast, at higher dye concentrations (15.0 and 20.0 ppm), the degradation efficiency significantly decreased. Removal efficiencies of approximately 46% and 22%, respectively, were achieved after 75 min (Fig. 9). These findings suggest that the degradation efficiency is inversely correlated with dye concentration under the given experimental conditions.

#### 3.10.2. Influence of photocatalyst weight

**Fig. 10:**
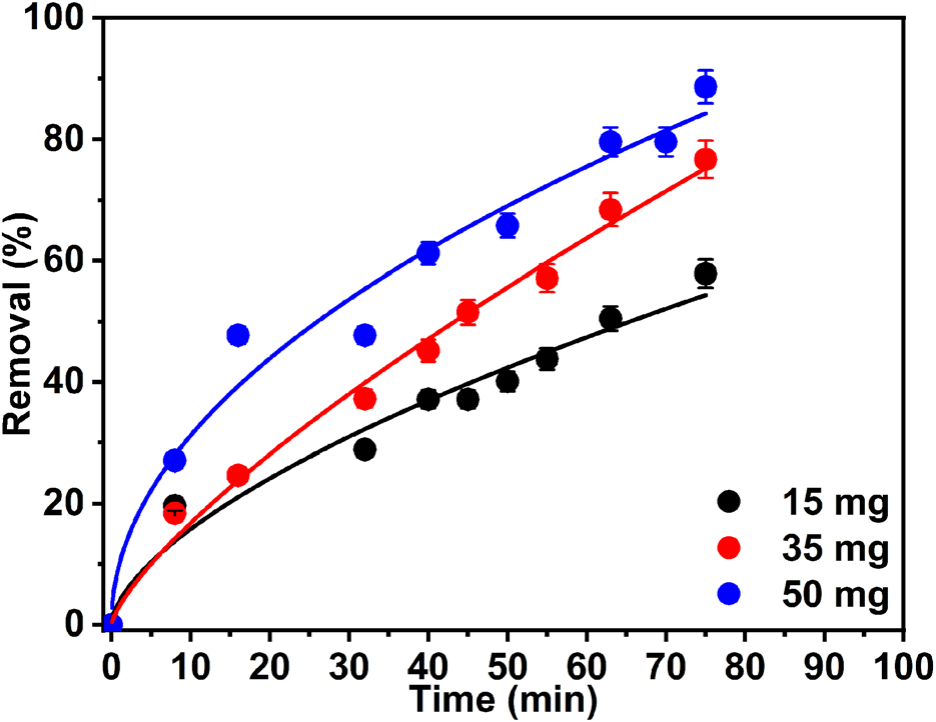
The effect of ZnO NPs (sample 2Z) dose on the photocatalytic degradation of 50 mL Rh 6G (10.0 ppm).

In this series of experiments, three distinct photocatalyst masses (sample 2Z) –15, 35, and 50 mg – were evaluated. The experiments were conducted at a pH of 6.5, utilizing 50 mL of a 10.0 ppm Rh 6G solution. The UV-Vis spectra, depicted in Fig. S8a–c, demonstrated the degradation of Rh 6G across the different photocatalyst masses. Variation in the extent of Rh 6G dye removal was observed for each tested photocatalyst mass. As illustrated in Fig. 10, the removal efficiency analysis indicated that the highest efficiency of 89% was achieved within 75 min using 50 mg of the photocatalyst. Consequently, this mass was identified as the optimal dosage for the effective removal of the dye at the specified concentration of 10 ppm.

#### 3.10.3. The influence of medium's pH

**Fig. 11:**
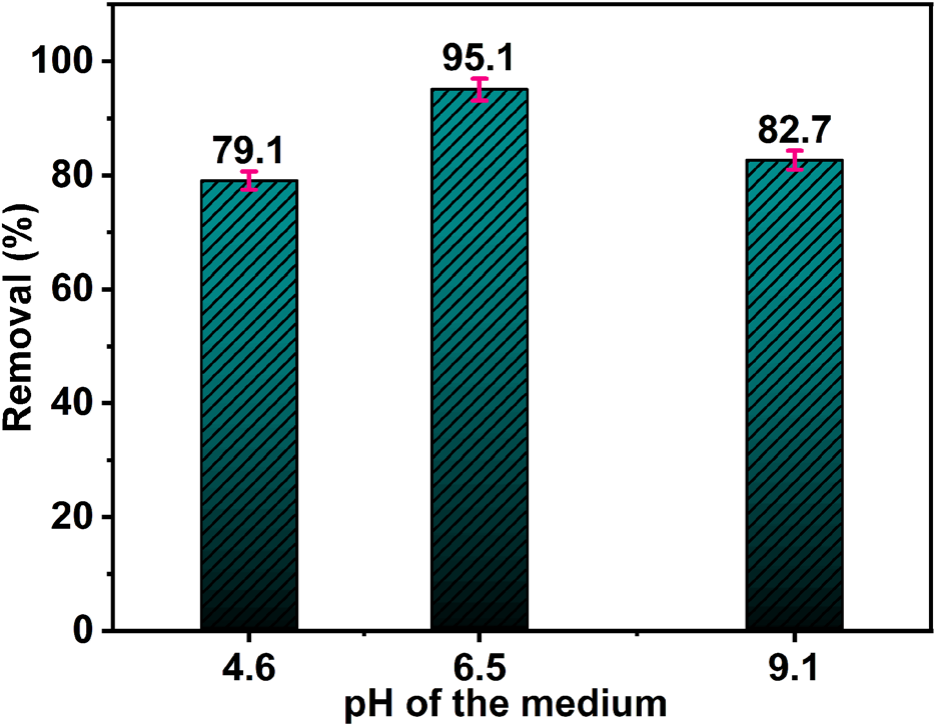
Effect of initial pH of the medium on the photocatalytic degradation of Rh 6G (5 mL, 5.0 ppm) using 50 mg of ZnO NPs (sample 2Z) and after 55 min exposure to sunlight.

In order to investigate the influence of the pH of the reaction medium on Rh 6G decolorizing, a set of experiments was performed at pH values of 4.6, 6.5, and 9.1. The used Rh 6G concentration in these experiments was 5.0 ppm and the ZnO NPs mass was 50 mg. The results shown in Fig. 11 reveal that the removal of Rh 6G dye using ZnO NPs is strongly influenced by the pH of the medium. The highest Rh 6G degradation of 95.1% was observed at pH 6.5 after 55 min exposure to sunlight. Accordingly, the near neutral medium (pH 6.5) is the most favorable medium for the Rh 6G degradation using ZnO NPs (sample 2Z). The interaction between Rh 6G and ZnO NPs is governed by several factors that are directly related to pH, including surface charge, adsorption efficiency, photocatalytic activity, and the stability of both the dye and the nanoparticles.

Here's possible explanations of why pH 6.5 is the optimum pH medium for the removal of Rh 6G dye using ZnO NPs. ZnO NPs typically have surface hydroxyl groups (–OH), and their behavior changes with pH. At pH 6.5, the surface of ZnO is slightly negatively charged, and Rh 6G (as a cationic dye) can easily interact with these surface groups *via* electrostatic forces. Electrostatic interaction between the positively charged dye and the negatively charged ZnO surface maximizes adsorption. This is critical for the removal process since the higher surface coverage leads to more effective photocatalytic degradation or adsorption of the dye. In contrast, at very acidic pH (4.6), ZnO particles may become positively charged, which could result in repulsion between the dye and the nanoparticles, reducing the adsorption, which significantly reduced the catalyst effectiveness.^55^ At higher pH, the ZnO surface may become excessively negative, reducing the efficiency of Reactive Oxygen Species (ROS) generation such as hydroxyl radicals (OH˙) under sunlight and hence hindering the overall catalytic process. Furthermore, ZnO NPs are typically more stable in mildly acidic to neutral conditions (pH around 6) than at extreme pH values. Extreme acidity (very low pH) can lead to the dissolution of ZnO particles, while highly alkaline conditions can lead to the formation of Zn(OH)_2_, which reduces the availability of active ZnO sites.

#### 3.10.4. Effect of interfering anions

**Fig. 12:**
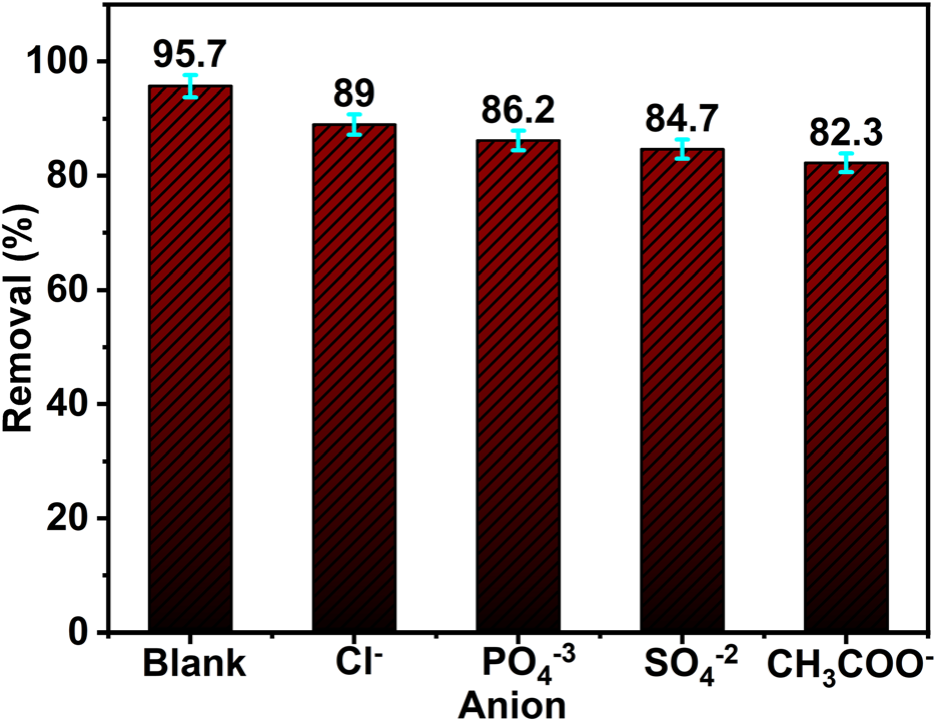
Effect of some inorganic anions on the photocatalytic degradation of 50 mL Rh 6G (5.0 ppm) using 50 mg of ZnO NPs (2Z) and after 75 min exposure to sunlight.

Industrial dye effluents commonly contain inorganic anions such as chloride (Cl^−^), phosphate (PO_4_^3−^), sulfate (SO_4_^2−^), and acetate (CH_3_COO^−^), which can adversely affect the efficiency of photocatalytic degradation processes.^56^ These anions contribute to colloidal instability, increase mass transfer resistance, and reduce the interaction between dye molecules and photocatalyst surfaces. In this context, a study was conducted by introducing 5 mM of each anion into a Rh 6G solution treated with ZnO NPs. The results (Fig. 12) revealed that chloride had a minimal inhibitory effect, causing less than a 7% decrease in degradation efficiency. However, phosphate and sulfate significantly suppressed photocatalytic activity by acting as electron–hole scavengers, generating less reactive radicals and blocking the active sites on ZnO, thereby hindering dye adsorption. Acetate ions demonstrated the most substantial inhibitory effect, with approximately a 13% reduction in Rh 6G degradation. These findings emphasize the critical impact of coexisting anions in wastewater, highlighting the need to consider background ionic composition when designing and optimizing photocatalytic systems for real-world water treatment applications.

#### 3.10.5. Kinetics studies

The kinetics of Rh 6G photocatalytic degradation were analyzed using the Langmuir–Hinshelwood (L–H) model, which describes the relationship between the degradation rate (*r*) of an organic compound and its concentration (*C*) at time *t*, as follows:^57^5
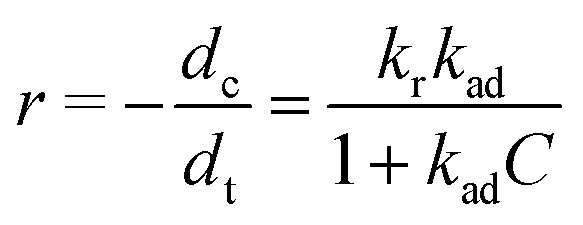
where *k*_r_ and *k*_ad_ represent the reaction rate constant and the adsorption equilibrium constant, respectively. Under the assumption that the initial concentration *C*_0_ is low, the denominator simplifies, and the system approximates a pseudo-first-order reaction. This leads to the linearized form:^58,59^6
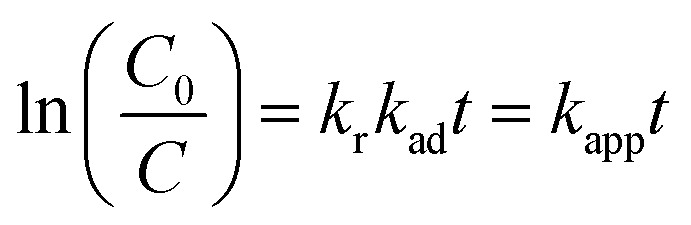
where *k*_app_ is the apparent first-order rate constant.

**Fig. 13:**
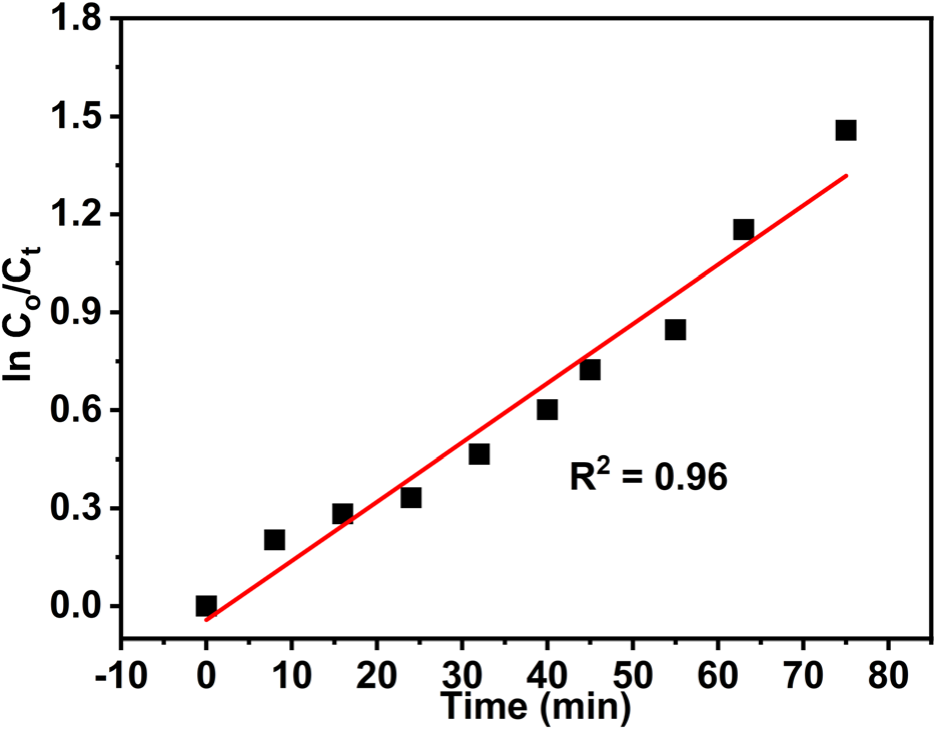
Kinetics of the photocatalytic degradation of Rh 6G (50 mL, 5.0 ppm) using 50 mg ZnO NPs as photocatalyst.

The value of *k*_app_ was determined from the slope of the linear plot of ln(*C*_0_/*C*) *versus t*. As shown in Fig. 13, the degradation of Rh 6G using ZnO NPs in aqueous solution followed pseudo-first-order kinetics, with a calculated rate constant of 0.018 min^−1^, confirming the suitability of the L–H kinetic model for this system.

#### 3.10.6. Reusability and stability of the photocatalyst

**Fig. 14:**
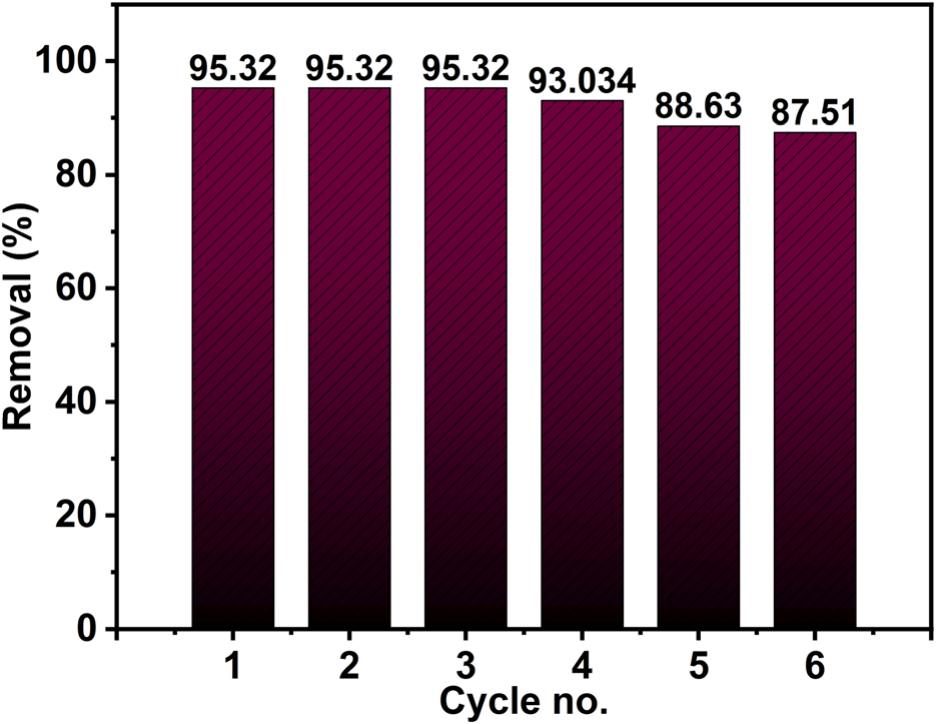
Recyclability test of ZnO NPs (2Z) for the removal of 50 mL Rh 6G (5.0 ppm) after 75 min exposure to sunlight.

The reusability of ZnO NPs (2Z) synthesized using *Capparis decidua* stems extract as a photocatalyst was systematically evaluated. Following the initial photocatalytic cycle, the photocatalyst was recovered, subjected to sequential washing with deionized water and ethanol, and subsequently dried overnight. The recycled photocatalyst was then employed in consecutive photocatalytic cycles to assess its stability and performance. The findings, as illustrated in Fig. 14, demonstrate a minimal decline in the photocatalytic efficiency of the ZnO NPs after six successive cycles, indicating a gradual reduction in activity with repeated use. This decrease in efficiency may be attributed to factors such as partial agglomeration of NPs, surface contamination, or loss of active sites during the recycling process. Nevertheless, the minimal reduction in performance highlights the robust stability and potential reusability of the biosynthesized ZnO NPs, making them a promising candidate for sustainable photocatalytic applications. Further optimization of the recovery and regeneration process could enhance their long-term efficiency and practical applicability.

**Fig. 15:**
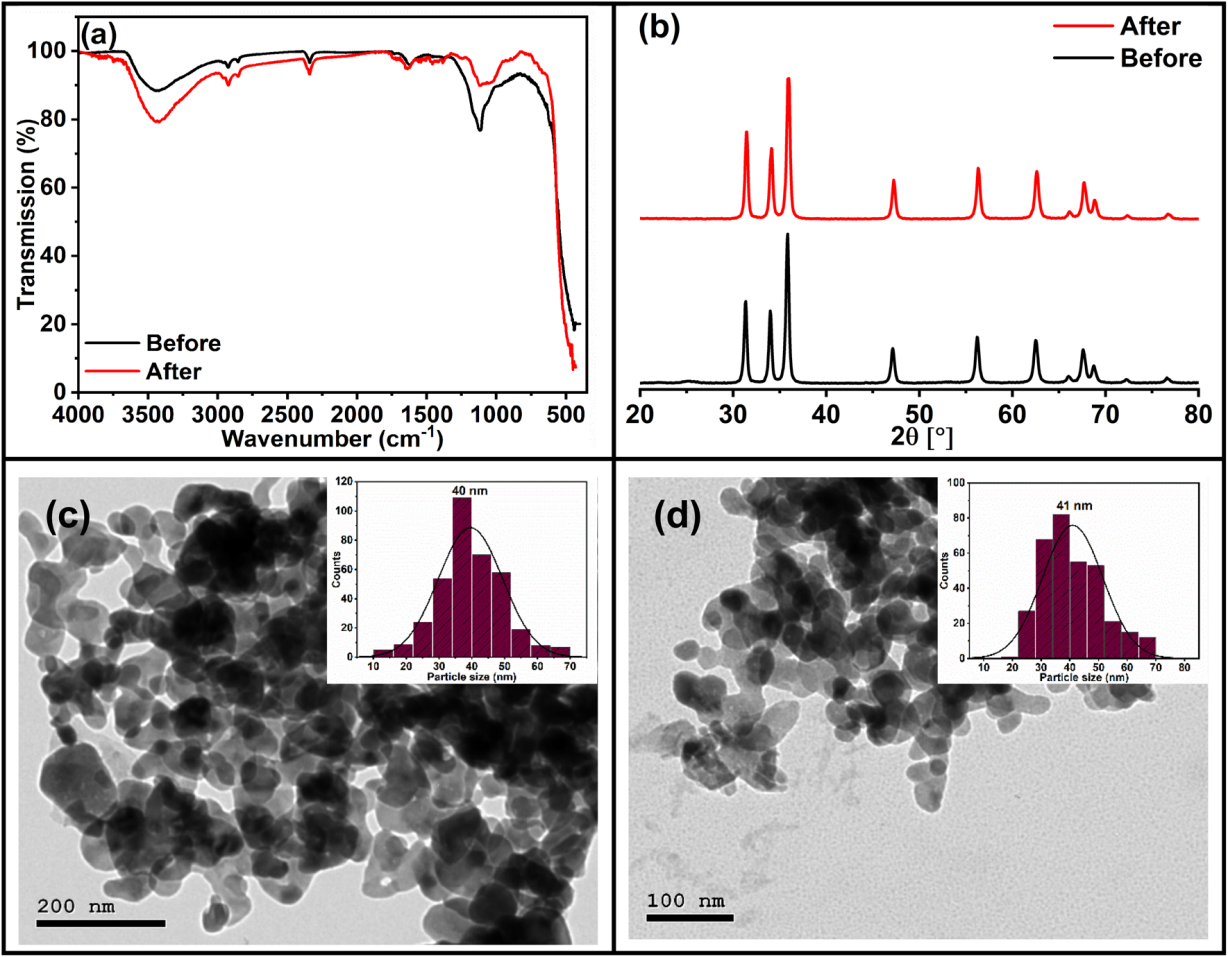
(a) FTIR spectra, (b) XRD powder patterns, and (c and d) TEM images of ZnO NPs (2Z) before and after six consecutive photocatalytic cycles of the Rh 6G degradation.

The stability of the photocatalyst was evaluated following recycling experiments through characterization using IR and XRD. The IR spectra and XRD patterns (Fig. 15a and b) of ZnO, obtained before and after the recycling experiments, indicate no detectable structural alterations. The results that suggest the preservation of the material's crystalline phase and functional groups. The absence of significant changes in the IR absorption bands and XRD diffraction peaks further confirms that no decomposition or phase transformation occurred during the photocatalytic process. Furthermore, TEM analysis was made to investigate potential morphological changes at the nanoscale. The TEM images (Fig. 15c and d) revealed that the particle morphology remained largely intact after six cycles, with no observable aggregation or structural collapse, confirming the physical stability of the nanoparticles.

These findings demonstrate that the photocatalyst exhibits excellent structural stability and recyclability under the tested conditions, which is a critical attribute for its practical application in sustainable photocatalytic systems. The consistent performance and structural integrity of ZnO highlight its potential as a durable and efficient photocatalyst for repeated use in environmental remediation or energy conversion processes.

The data presented in Table 4 clearly demonstrates the competitive performance of the ZnO photocatalyst developed in this study. Despite using a relatively simple ZnO system without complex doping or composite structures, the catalyst achieved a high Rh 6G removal efficiency of 96% under sunlight irradiation within 75 min, surpassing or closely matching many previously reported systems that often rely on UV light or complex modifications (*e.g.*, PPy–ZnO composite). In contrast to some entries that required high catalyst doses or extended reaction times, the current study achieved high degradation efficiency under milder conditions, confirming enhanced photocatalytic performance. This can be attributed to the optimized synthesis approach and the morphological features of the ZnO NPs used. Furthermore, the use of *Capparis decidua* in the synthesis introduces a novel, eco-friendly, and sustainable component not previously reported in the literature for photocatalysis, reinforcing the novelty and green aspect of this work.

**Table 4:** Comparison of the photocatalytic performance of ZnO NPs with previously reported photocatalysts for Rh 6G degradation under different irradiation conditions

Entry	Photocatalyst	Dose (mg)	Removal condition	Source of irradiation	Removal (%)	Ref.
(1)	rGO/ZnO/Fe_2_O_3_	20	50 mL of Rh 6G (10 ppm), 240 min	Visible light	37.0 ± 2.9	35
(2)	Polypyrrole/ZnO composites	14	5 mL of 1 mM Rh 6G, 390 min	Halogen light	90	60
(3)	Citrate stabilized ZnO	100	10, 20, and 30 ppm of Rh 6G, 150 min	Visible light	53.7, 20.85, and 11.0	53
(4)	ZnO	30	12 ppm Rh 6G, 180 min	UV light	68.68	54
(5)	PPy-2% ZnO	30	12 ppm Rh 6G, 6 min	UV light	98.19	54
(6)	PbFe_12_O_19_ NPs	1–5	20 mL Rh 6G (5–20 ppm, 20–80 min	Sunlight	>95	37
(7)	MgO	5	10 ppm (250 mL) of Rh 6G, 180 min	UV light, visible light	92.62, 38.71	61
(8)	ZnO	50	50 mL (5 ppm) of Rh 6G, 75 min	Sunlight	96	Current study

#### 3.10.7. Mechanism of photodegradation

Upon exposure to sunlight, ZnO generates an electron–hole pair (e_CB_^−^/h_VB_^+^), initiating a series of photocatalytic processes. These reactions predominantly occur on the surface of ZnO, where the photo-generated electrons and holes are trapped, facilitating surface-mediated redox reactions.^62^ The interaction between the electrons and adsorbed oxygen results in the formation of superoxide radicals (O_2_˙^−^), which can function as either oxidants or reductants. In aqueous media, hydroxyl radicals (˙OH) are produced through the reaction of holes with adsorbed H_2_O or hydroxide groups (Fig. S9). These hydroxyl radicals are highly potent oxidants, capable of degrading a wide range of organic pollutants.^63^ The reactive species produced during ZnO photocatalysis, including h_VB_^+^, e_CB_^−^, ˙OH, O_2_˙^−^, ˙O_2_H, H_2_O_2_, ^1^O_2_ play a critical role in the degradation of pollutants under investigation. These species participate in complex redox reactions, leading to the breakdown of organic molecules into smaller, less harmful compounds, ultimately contributing to environmental remediation. The efficiency of this process is influenced by factors such as surface area, crystallinity, and the presence of defect sites on the ZnO surface, which can enhance the trapping and utilization of charge carriers.

Reactive oxygen species (ROS) play a pivotal role in photocatalytic processes, serving as key oxidizing agents responsible for the breakdown of organic contaminants.^56^ In this study, the photocatalytic degradation of Rh 6G was investigated using specific scavengers to identify the predominant reactive species involved in the degradation mechanism. Isopropanol, Na_2_EDTA, and benzoquinone were employed as scavengers for hydroxyl radicals (˙OH), photogenerated holes (h^+^), and superoxide radicals (O_2_˙^−^), respectively.^64^ The introduction of these scavengers led to a notable decline in degradation efficiency, reducing it to 75%, 74%, and 9%, respectively. The relatively moderate decrease observed with isopropanol and Na_2_EDTA suggests that both ˙OH radicals and holes contribute to the photocatalytic process, albeit to a lesser extent. In contrast, the significant suppression of activity in the presence of benzoquinone highlights the dominant role of superoxide radicals in the degradation pathway of Rh 6G. These results indicate that the generation and involvement of O_2_˙^−^ play a critical role in the overall photocatalytic mechanism. It is likely acting as the primary oxidative species responsible for Rh 6 G molecule breakdown followed by ˙OH and h^+^.

## 4. Conclusions

This study demonstrates the successful green synthesis of ZnO NPs using *Capparis decidua* stem extract as an effective coordinating, capping, and stabilizing agent. The extract concentration significantly influenced the structural and photocatalytic properties of the nanoparticles. The highest concentration produced smaller spherical particles (about 24 nm), with largest surface area of 32.9 m^2^ g^−1^, and greater pore volume of 0.039 cm^3^ g^−1^. On the contrary, the lowest concentration yielded ZnO with largest spherical particles (40 nm), smallest surface area of 2.2 m^2^ g^−1^ and pore volume of 0.008 cm^3^ g^−1^. The ZnO sample synthesized with the lowest extract concentration (2Z) achieved the highest Rh 6G degradation efficiency (96%) under sunlight at pH 6.5 within 75 min, following first-order kinetics (*k* = 0.018 min^−1^). This enhanced performance could be attributed to its wider bandgap (3.67 eV) which may enhance the generation of reactive oxygen species (primarily superoxide radicals (O_2_˙^−^) and supported by ˙OH and h^+^ species) and reduce electron–hole recombination. In contrast, the sample obtained using the highest extract concentration (10Z) exhibited the narrowest bandgap (3.02 eV) and lowest photocatalytic efficiency, suggesting that excessive organic content may introduce defects or hinder charge separation. Overall, the results confirm *Capparis decidua* as a sustainable precursor for producing efficient ZnO-based photocatalysts and emphasize the need to balance surface and electronic properties to achieve optimal photocatalytic activity for environmental applications.

## Conflicts of interest

There are no conflicts to declare.

## Supplementary Material

RA-015-D5RA05878C-s001

## Data Availability

The datasets generated and/or analyzed during the current study are available from the corresponding author on reasonable request. All relevant data supporting the findings of this study are included within the article and its supplementary information (SI) files. Supplementary information is available. See DOI: https://doi.org/10.1039/d5ra05878c.
