# Supplementary material for: Sustainable production of ZnO nanoparticles via capparis decidua stem extract for efficient photocatalytic Rh 6G dye degradation
Source: RSC Adv. 2025 Nov 27;15(55):46890–907. doi: 10.1039/d5ra05878c (PMC12659785; doi:10.1039/d5ra05878c)
Supplement: RA-015-D5RA05878C-s001 [file RA-015-D5RA05878C-s001.pdf]

Supplementary Information (SI) for RSC Advances.
This journal is © The Royal Society of Chemistry 2025

Sustainable Production of ZnO Nanoparticles via *Capparis Decidua* Stem Extract for Efficient Photocatalytic Rh 6G Dye Degradation

Asmaa M. Sayed[a], Fawzy M. Salam[a], Hanaa K. Galal[a], and Mohamed I. Said[b,*]

[a] **Department of Botany and Microbiology, Faculty of Science, Assiut University, Egypt**

[b] **Department of Chemistry, Faculty of Science, Assiut University, Egypt**

*** Corresponding author**

e-mail: *moh_chem1@yahoo.com*

mohamedali123@aun.edu.eg

Keywords

Capparis Decidua; Green Synthesis; Plant Extract; Zinc Oxide NPs; Optical Properties; Rhodamine 6G Removal.

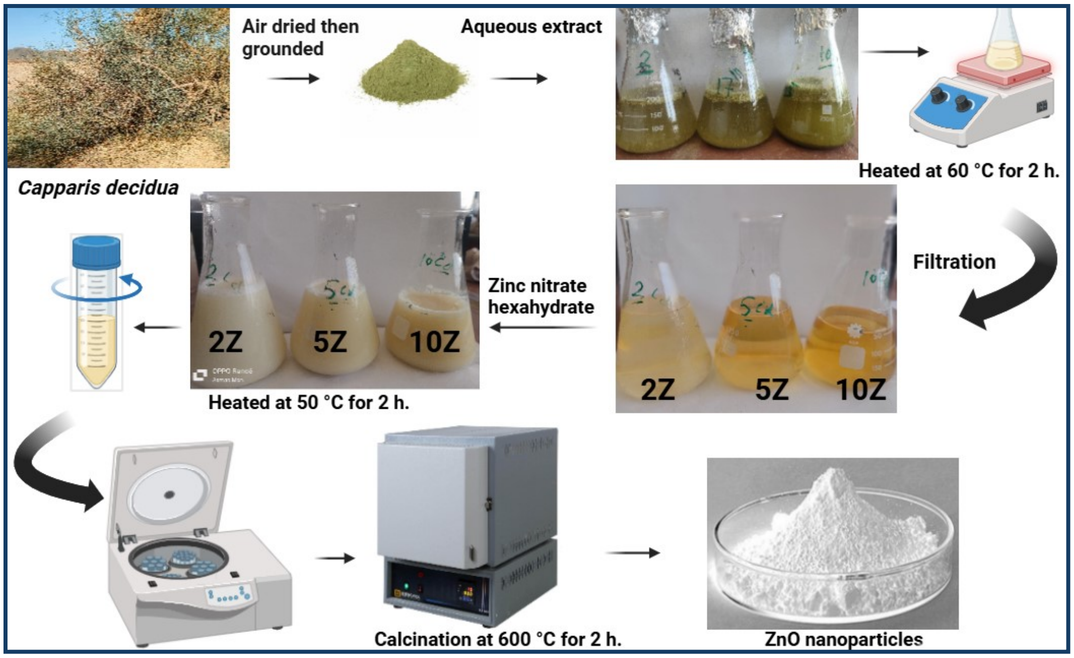

Fig. S1 Schematic illustration for the green synthesis of ZnO NPs.

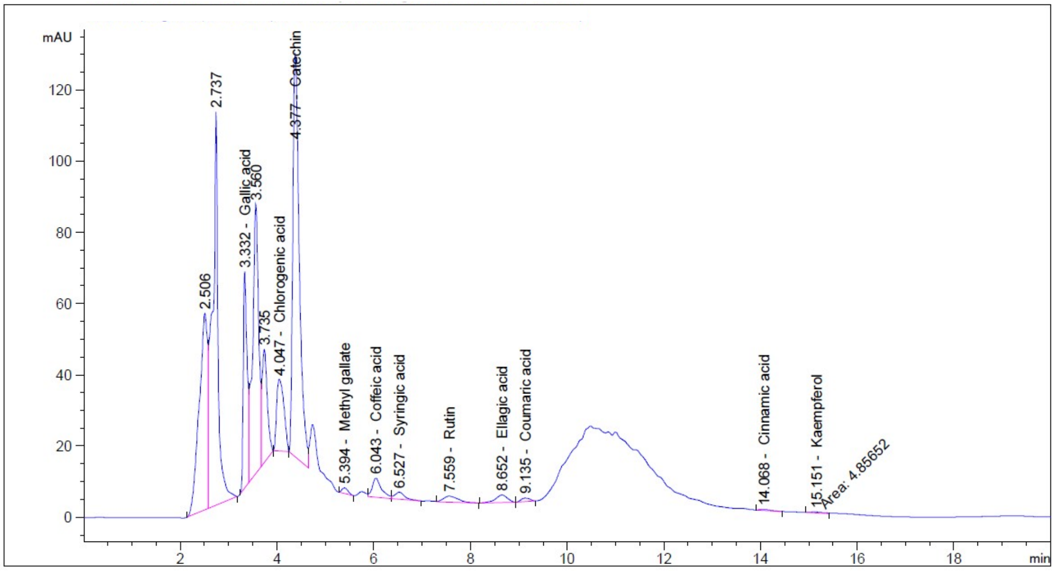

Figure S2. HPLC chromatogram of the *Capparis Decidua extract.*

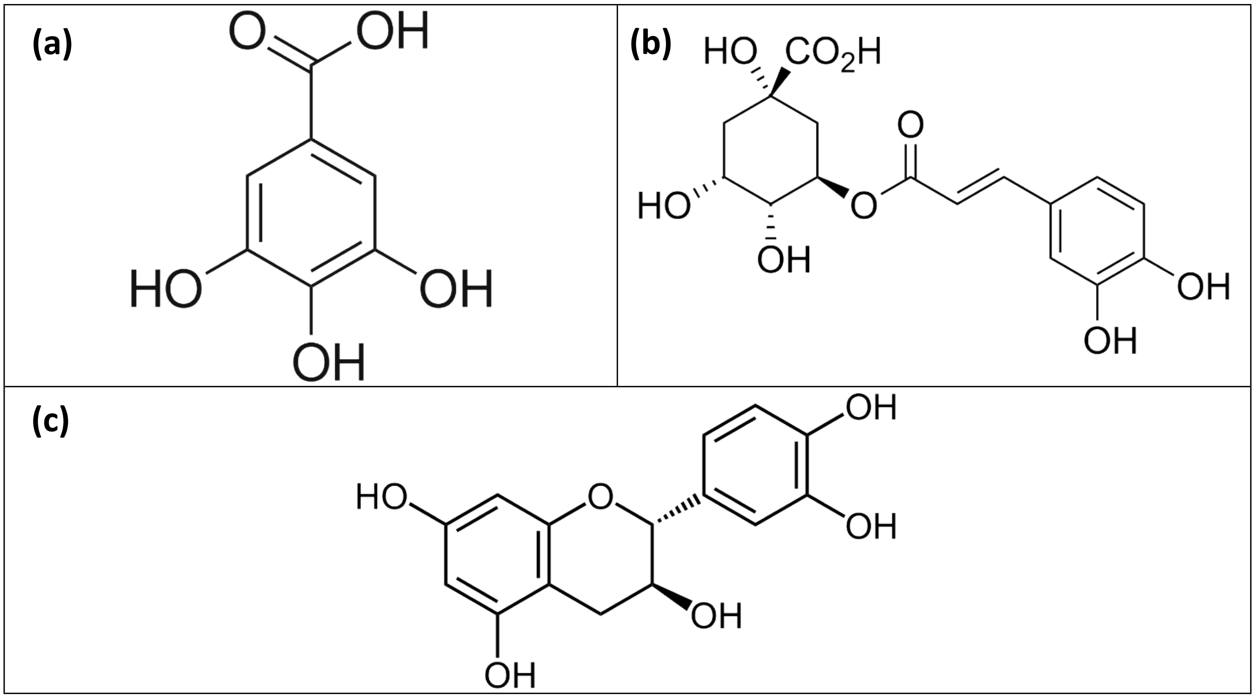

Figure S3. Chemical structure of a) Gallic acid, b) Chlorogenic acid, and c) Catechin.

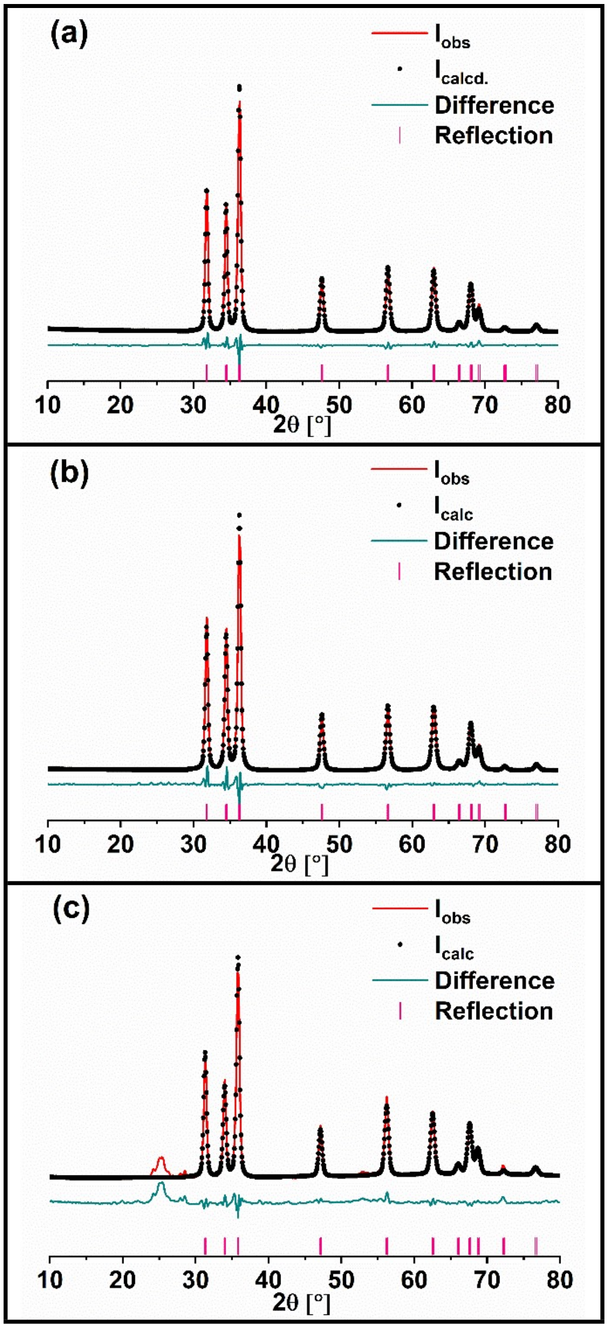

Fig. S4 Rietveld analysis for the XRD patterns of the three ZnO samples prepared using different plant extract concentrations: a) 2Z, b) 5Z and c) 10Z.

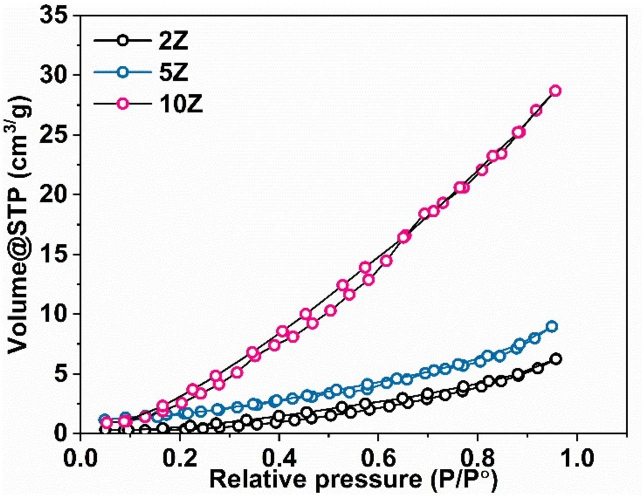

Fig. S5 Nitrogen adsorption-desorption isotherms of ZnO NPs prepared using different plant extracts.

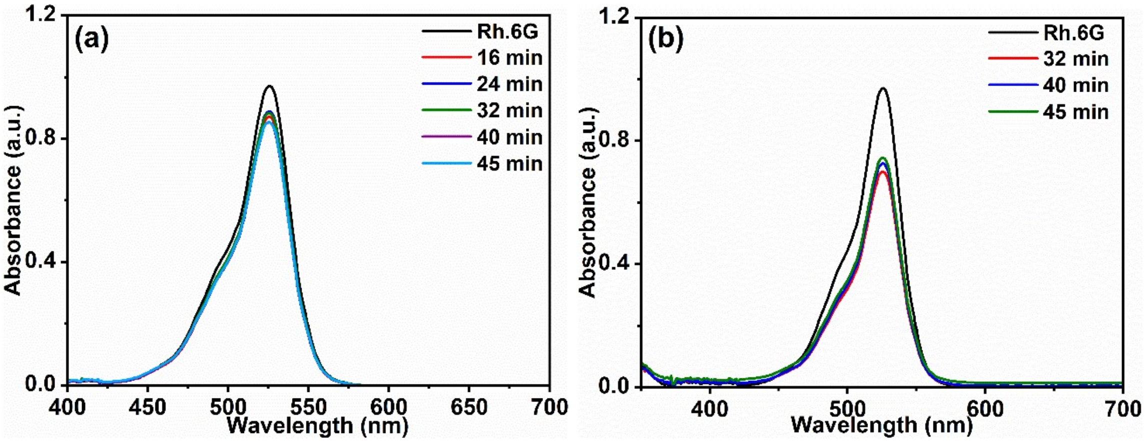

Fig. S6 UV-Vis spectra of a) Rh 6G subjected to sunlight for 45 min, and b) dark part experiment for Rh 6G removal using 50 mg ZnO NPs.

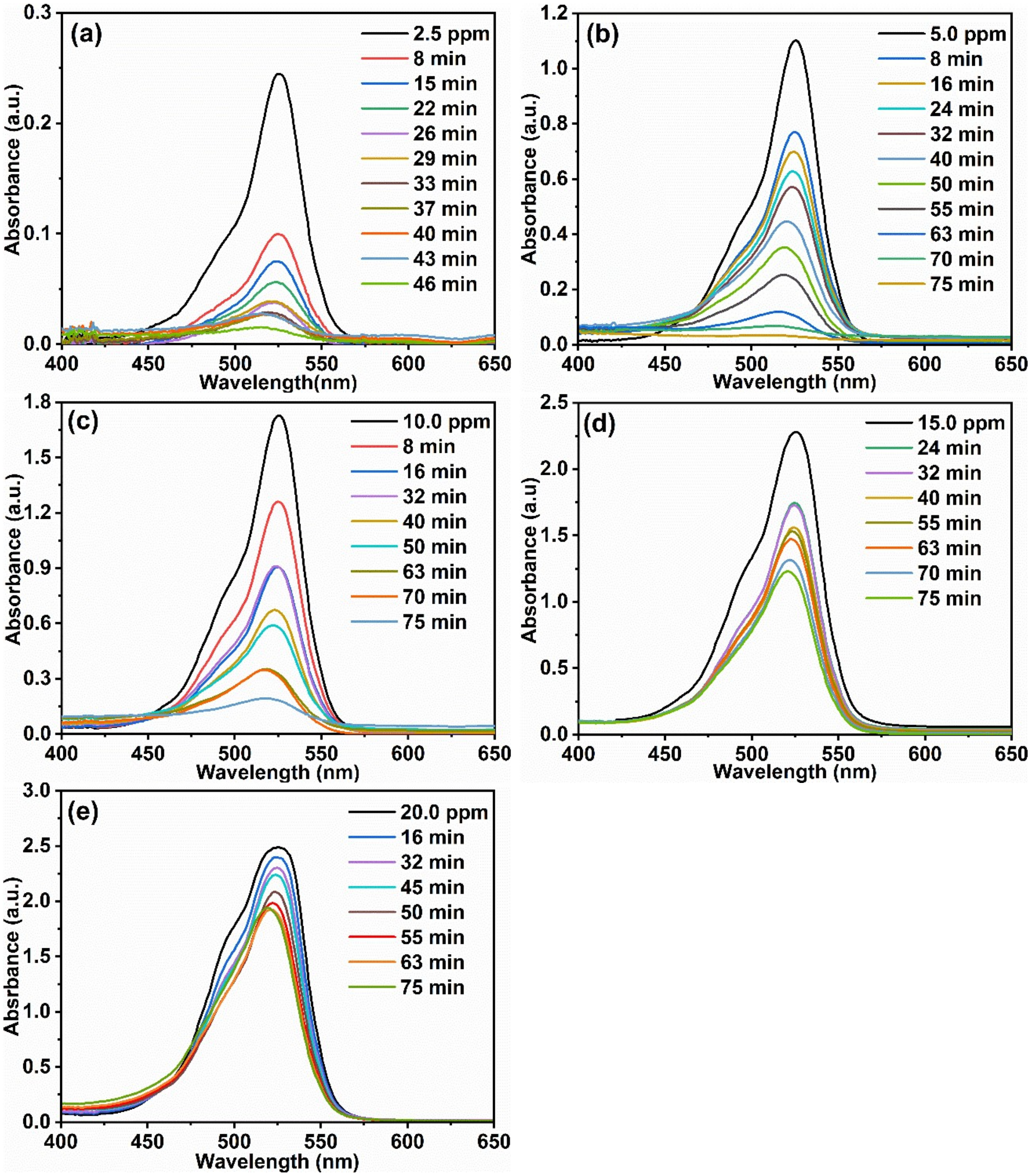

Fig. S7 Uv-Vis spectra showing the effect of dye concentration on the degradation of 50 mL Rh 6G while using 50 mg of ZnO NPs (sample 2Z) as photocatalyst.

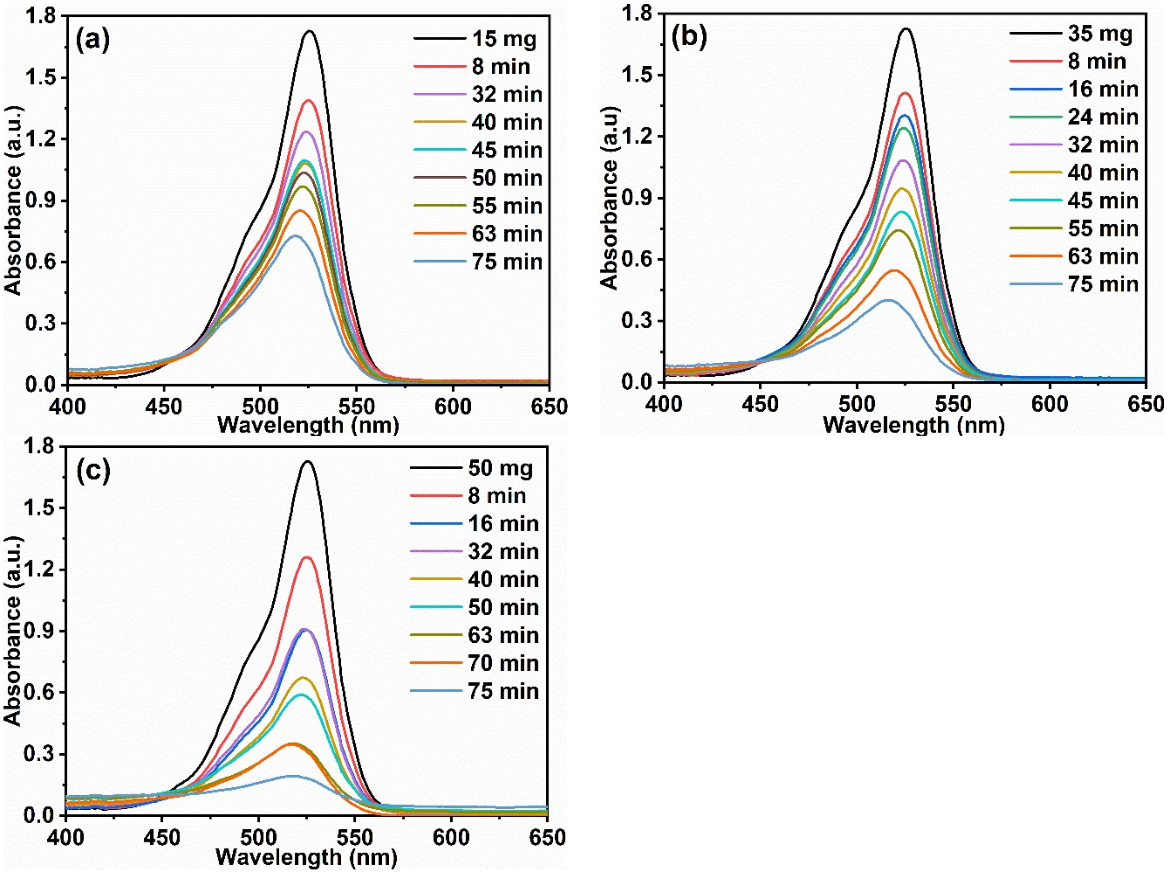

Fig. S8 Uv-Vis spectra showing the effect of ZnO NPs (sample **2Z**) dose on the photocatalytic degradation of 50 mL Rh 6G (10.0 ppm).

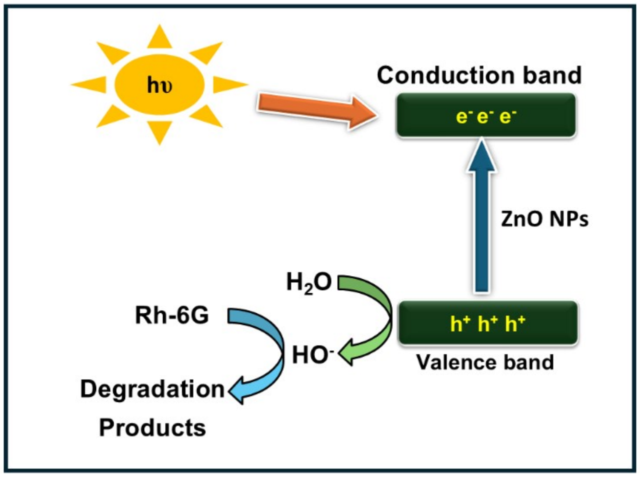

Fig. S9 Production of hydroxyl radical (•OH) occurs in aqueous medium.
